# β1-Adrenergic Receptor Contains Multiple IA^k^ and IE^k^ Binding Epitopes That Induce T Cell Responses with Varying Degrees of Autoimmune Myocarditis in A/J Mice

**DOI:** 10.3389/fimmu.2017.01567

**Published:** 2017-11-20

**Authors:** Rakesh H. Basavalingappa, Chandirasegaran Massilamany, Bharathi Krishnan, Arunakumar Gangaplara, Rajkumar A. Rajasekaran, Muhammad Z. Afzal, Jean-Jack Riethoven, Jennifer L. Strande, David Steffen, Jay Reddy

**Affiliations:** ^1^School of Veterinary Medicine and Biomedical Sciences, University of Nebraska-Lincoln, Lincoln, NE, United States; ^2^Department of Medicine, Division of Cardiology, Medical College of Wisconsin, Milwaukee, WI, United States; ^3^Center for Biotechnology, University of Nebraska-Lincoln, Lincoln, NE, United States

**Keywords:** autoimmunity, β1-adrenergic receptor, myocarditis, mouse model, T cells

## Abstract

Myocarditis/dilated cardiomyopathy (DCM) patients can develop autoantibodies to various cardiac antigens and one major antigen is β1-adrenergic receptor (β_1_AR). Previous reports indicate that animals immunized with a β_1_AR fragment encompassing, 197–222 amino acids for a prolonged period can develop DCM by producing autoantibodies, but existence of T cell epitopes, if any, were unknown. Using A/J mice that are highly susceptible to lymphocytic myocarditis, we have identified β_1_AR 171–190, β_1_AR 181–200, and β_1_AR 211–230 as the major T cell epitopes that bind major histocompatibility complex class II/IA^k^ or IE^k^ alleles, and by creating IA^k^ and IE^k^ dextramers, we demonstrate that the CD4 T cell responses to be antigen-specific. Of note, all the three epitopes were found also to stimulate CD8 T cells suggesting that they can act as common epitopes for both CD4 and CD8 T cells. While, all epitopes induced only mild myocarditis, the disease-incidence was enhanced in animals immunized with all the three peptides together as a cocktail. Although, antigen-sensitized T cells produced mainly interleukin-17A, their transfer into naive animals yielded no disease. But, steering for T helper 1 response led the T cells reacting to one epitope, β_1_AR 181–200 to induce severe myocarditis in naive mice. Finally, we demonstrate that all three β_1_AR epitopes to be unique for T cells as none of them induced antibody responses. Conversely, animals immunized with a non-T cell activator, β_1_AR 201–220, an equivalent of β_1_AR 197–222, had antibodies comprising of all IgG isotypes and IgM except, IgA and IgE. Thus, identification of T cell and B cell epitopes of β_1_AR may be helpful to determine β_1_AR-reactive autoimmune responses in various experimental settings in A/J mice.

## Introduction

Myocarditis is one major cause of dilated cardiomyopathy (DCM) that can lead to heart failure in young adults in the developed countries. Approximately, half of those affected with DCM undergo heart transplantation due to lack of effective therapeutic options (1–4). Clinically, majority of DCM patients (up to 80%) are designated to be idiopathic DCM (IDCM), and the detection of heart infiltrates in those affected suggests that immune dysfunction may be an underlying mechanism in the DCM pathogenesis (5–8). Growing evidence suggests that autoimmune responses may be an important trigger, since DCM patients can show autoantibodies for various antigens such as cardiac myosin, cardiac troponin I, adenine nucleotide translocator 1 (ANT), β1-adrenergic receptor (β_1_AR), and branched chain alpha-ketoacid dehydrogenase (BCKD) (9–12). For example, β_1_AR-reactive antibodies can be detected in 26–60% of IDCM patients as compared to ischemic cardiomyopathy (10–13%) or healthy controls (<10%) (6, 7, 13). While, DCM patients can show antibody reactivity to cardiac myosin heavy chain-α (Myhc) (66%) and troponin-I (50%), up to 80% of the end-stage IDCM patients or more than 90% of patients using left ventricular assist device were shown to be positive for antibodies to β_1_AR (6, 7). These observations suggest that β_1_AR may be a major autoantigen in the initiation and progression of DCM.

β-adrenergic receptors are classified into four subtypes with β_1_AR being found primarily in the heart, whereas β_2_AR is expressed in the lung, kidney, blood vessels and heart, and β_3_AR-expression occurs primarily in the adipose tissue (14, 15). Although, mRNA and protein data are lacking, existence of β_4_AR has been reported, which appears to represent the low-affinity state of β_1_AR (16). Belonging to G-protein-coupled receptors, β_1_AR contains three each of extracellular (EC) and intracellular loops. The natural ligands of β_1_AR namely, adrenaline and nor-adrenaline trigger activation of cyclic adenosine monophosphate (cAMP) and protein kinase A leading to influx of calcium (Ca^2+^) through L-type Ca^2+^ channel and cardiac contractility (17, 18). Similar events can be expected under conditions of autoimmunity as might occur with the presence of β_1_AR-reactive autoantibodies. In fact, antibodies recognizing epitopes localized within the EC loops, particularly EC loop II (β_1_AR 197–222), can agonistically trigger Ca^2+^ release and cAMP activation, without affecting interaction of β_1_AR with its natural ligands (6, 19). Such antibodies have been detected in DCM patients indicating their pathological significance (13, 20).

Experimentally, monthly immunizations for 6–18 months with β_1_AR 197–222 can result in the production of antibodies in various species such as rabbits, rats, and mice, and the immunized animals can develop features of cardiomyopathy during the course of ~1 year with little or no myocarditis (21–25). While, an assumption made in these studies that a single epitope of β_1_AR can act as an immunogen in various species despite the major histocompatibility complex (MHC) alleles to be different was found valid, identification of other potential immunodominant epitopes, if any, was not explored. Likewise, it was unknown whether β_1_AR possesses T cell epitopes and contributes to disease. This is particularly important because, for B cells to produce antibodies of different isotypes, T cell help is critical, and identification of T cell epitopes may thus provide another layer of evidence for pathologic importance of β_1_AR in the DCM pathogenesis. To this end, we sought to characterize the T cell epitopes of β_1_AR in A/J mice that are highly susceptible to lymphocytic myocarditis leading us to identify several epitopes that induce differential T cell and/or antibody responses. By testing for myocarditogenicity, we noted that the antigen-primed T cells could induce severe myocarditis in naive recipients, but the T cells needed to produce primarily, T helper (Th) 1 cytokines, in addition to Th17 cytokines.

## Materials and Methods

### Mice

Six- to eight-week-old, female A/J mice (H-2^a^) procured from the Jackson Laboratory (Bar Harbor, ME, USA) were maintained in accordance with the institutional guidelines of the University of Nebraska-Lincoln. Approval for animal studies was granted by the Institutional Animal Care and Use Committee, University of Nebraska-Lincoln, Lincoln, NE, USA (protocol #: 1398). Euthanasia was performed using a carbon dioxide chamber as recommended by the Panel on Euthanasia, the American Veterinary Medical Association.

### Peptide Synthesis

An overlapping peptide library that included a total of 46 peptides of 20-mers, except one peptide, β_1_AR 451–466 (16-mer) was created. In addition to these, bovine ribonuclease (RNase) 43–56 (VNTFVHESLADVQA), biotinylated hen egg lysozyme (HEL) 46–61 (YNTDGSTDYGILQINSR) (Neopeptide, Cambridge, MA, USA), and moth cytochrome *c* (MCC) 82–103 (FAGLKKANERADLIAYLKQATK) (GenScript, Piscataway, NJ, USA) were synthesized by 9-fluorenylmethyloxycarbonyl chemistry. Where indicated, acetylated β_1_AR (β_1_AR_Ac_) that contain acetyl group at the N-terminal end were used. All peptides were high-performance liquid chromatography-purified (>90%), and their identities were confirmed by mass spectroscopy. Ultrapure water was used to dissolve peptides and stored at −20°C.

### Immunization Procedures

Peptide emulsions were prepared in complete Freund’s adjuvant (CFA) containing *Mycobacterium tuberculosis* H37RA extract (Difco Laboratories, Detroit, MI, USA) to a final concentration of 5 mg/ml. To induce disease, animals were immunized twice s.c., in inguinal and sternal regions on days 0 and 7, and all animals received pertussis toxin (PT, List Biological Laboratories, Campbell, CA, USA; 100 ng/mouse) i.p., on days 0 and 2 after the first immunization (26–29). In pooled settings, 50 µg of each peptide was used, and when peptides were used individually, 100 µg was used per animal. To measure T cell responses in some experiments, a single dose of peptide emulsions were used.

### Histology

Hearts, and non-cardiac tissues (brain, lung, liver, and kidney) collected at termination on day 21 postimmunization were fixed by immersing in 10% phosphate-buffered formalin (26, 28). Longitudinal tissue layers were cut from hearts, and also from non-cardiac tissues. Serial sections with 5 µm thickness were then obtained and stained with hematoxylin and eosin (H&E). Analysis was performed by a board-certified pathologist blinded to treatment. After ascertaining the inflammatory changes, total number of inflammatory foci were determined by sections with the largest number of foci or by adding non-overlapping foci across sections as reported previously (26, 28, 30, 31).

### Immunohistochemistry (IHC)

To detect T cells in hearts, formalin-fixed paraffin-embedded tissue sections were stained with rabbit anti-mouse CD3 (clone SP7, 1:100, Abcam, Cambridge, MA, USA), rabbit anti-mouse CD4 (polyclonal, 1:100, Novus Biologicals, Littleton, CO, USA), and rabbit anti-mouse CD8 (clone EP1150Y, 1:100, Novus Biologicals) or their isotype controls. For non-T cells namely, neutrophils, macrophages, and B cells, rat anti-mouse Ly6G (clone 1A8, 1:250, Leinco Technologies, Fenton, MO, USA), rabbit anti-mouse CD11b (clone EPR1344, 1:3,500, Abcam) (30), and rat anti-mouse CD19 (clone 6OMP31, 1:1000, Thermo Fisher Scientific, San Diego, CA, USA) and their isotype controls were used, respectively. In brief, after deparaffinization, rehydration, and blockade of endogenous peroxidase activity, antigen retrieval was performed by treating the sections with 10 mM sodium citrate buffer (pH 6.0) in a water bath at 98°C for 15–40 min or using a pressure cooker. Sections were then blocked with 5% non-fat dry milk for 30 minutes, incubated with primary antibodies (Abs) at 4°C overnight; followed by incubation with horseradish peroxidase (HRP)-conjugated, goat anti-rabbit IgG or goat anti-rat IgG (Abcam) as secondary antibodies for 2 h at room temperature (RT). Diaminobenzoic acid was used as a substrate for color development before counterstaining with hematoxylin (26, 30). For quantitative analysis (T cells: CD3^+^, CD4^+^, and CD8^+^; and non-T cells: Ly6G^+^, CD11b^+^, and CD19^+^) in the heart, five random areas were selected from the representative sections and nuclear staining was confirmed using nuclear V9 software (Aperio Technologies, Vista, CA, USA). Cells positive for each marker were then enumerated and normalized to 1 mm^2^ area using Aperio ImageScope Analysis Software (Leica Biosystems, MN, USA) as we have described previously (30).

### Echocardiography and Image Analysis

Transthoracic echocardiography was performed in anesthetized animals immunized with or without a cocktail of β_1_AR_Ac_ 171–190, β_1_AR_Ac_ 181–200, and β_1_AR_Ac_ 211–230 on day 20. Scanning was performed by a research sonographer, blinded to the study groups, using a commercially available echocardiography system (Vivid 7, General Electric, Wauwatosa, WI, USA) with an M12-L linear array transducer as we reported previously (30). The mice were anesthetized with isoflurane and images were captured in the short-axis view at the mid-left ventricle (LV) level, verified by the presence of prominent papillary muscles. Three consecutive cardiac cycles, defined from the peak of one R wave to the peak of the following R wave were measured and used for analysis as previously reported (30). In brief, linear measurements were performed using the M-mode view to assess width of the intraventricular septum at diastole and the internal diameter of the LV at diastole and systole. The Teichholz formula {LV volume = [7/2.4 + left ventricular internal diameter (LVID)] × LVID^3^} was used to calculate end-diastolic and end-systolic volumes (30). The relative wall thickness (RWT) was calculated as the ratio of 2 × LV posterior wall thickness and LV internal diameter at end-diastole. The RWT normal range is between 0.32 and 0.42. Total body surface area (BSA) was calculated using the Meeh’s formula (BSA = 10 × [weight]^2/3^) (32). All measurements were indexed to BSA to account variation in size.

### T Cell Proliferation Assay

At termination, lymph nodes (maxillary, mandibular, axillary, inguinal, and popliteal) and spleens were harvested from immunized animals to prepare single cell suspensions. Similarly, splenocytes were prepared from naive animals. After lysing the erythrocytes and washing, cell pellets were suspended in RPMI medium containing 10% fetal bovine serum, 1 mM sodium pyruvate, 4 mM l-glutamine, 1 × each of non-essential amino acids and vitamin mixture, and 100 U/ml penicillin–streptomycin (Lonza, Walkersville, MD, USA; hereafter called growth medium). In some experiments, CD4 T cells and CD8 T cells were enriched to a purity of ~95% by negative selection based on magnetic separation using IMAG (BD Biosciences, San Jose, CA, USA) (31). To stimulate CD4 and CD8 T cells, syngeneic naive irradiated splenocytes loaded with peptides were used as antigen-presenting cells at a ratio of 1:1. Cells were stimulated at a density of ~5 × 10^6^ cells/ml in triplicates with or without the immunizing peptides (0–100 µg/ml) in growth medium. RNase 43–56 or HEL 46–61 were used as irrelevant controls. After 2 days, cells were pulsed with tritiated ^3^[H]-thymidine (1 μCi/well; MP Biomedicals, Santa Ana, CA, USA), and 16 h later, proliferative responses were measured as counts per minute (cpm) using a Wallac liquid scintillation counter (Perkin Elmer, Waltham, MA, USA) (26, 28, 30).

### MHC Class II-Binding Assay

A/J mice express two MHC class II alleles, IA^k^ and IE^k^ (33, 34), and to determine the affinities of β_1_AR peptides, soluble IA^k^ and IE^k^ monomers were expressed (34, 35). In brief, the β-chain in each of IA^k^ and IE^k^ constructs contain sequence for class II-associated invariant-chain peptide 88–102 (VSQMRMATPLLMRPM) linked with thrombin cleavage site (34, 35). After expressing in the baculovirus, soluble IA^k^ and IE^k^ monomers were treated with thrombin (20 U/mg; Novagen, Madison, WI, USA) to release class II-associated invariant-chain peptide leading us to obtain empty IA^k^ and IE^k^ molecules. Reaction mixtures were prepared to include empty IA^k^ or IE^k^ monomers (0.35 µg), competitor peptides (β_1_AR_Ac_ 171–190, β_1_AR_Ac_ 181–200, β_1_AR_Ac_ 201–220, and β_1_AR_Ac_ 211–230) (0.00001–100 µM), and constant amounts of the biotinylated reference peptides HEL 46–61 (for IA^k^) or MCC 82–103 (for IE^k^) (1 µM) in a buffer containing 50 mM sodium phosphate pH 7.0, 100 mM sodium chloride, 1 mM EDTA, and 1 × protease inhibitor (Sigma-Aldrich, St. Louis, MO, USA) (36–38). After incubating overnight at RT, the mixtures were transferred to 96-well white fluorescence plates coated with anti-IA^k^ (clone 10-2.16, BioXcell, West Lebanon, NH, USA), and anti-IE^k^ (clone M5/114, BioXcell) Abs (10 µg/ml) in 0.2 M sodium phosphate buffer, pH 6.8, blocked with 2% bovine casein; and the plates were incubated on a rocker at RT for 1 h. Finally, after adding 100 µl of europium-labeled streptavidin (0.1 µg/ml) and dissociation-enhanced lanthanide fluoroimmunoassay (DELFIA) enhancement solution (Perkin Elmer) sequentially, fluorescence intensities were measured at excitation/emission wavelengths of 340/615 nm using a Victor Multilabel Plate Reader (Perkin Elmer). The IC_50_ values were determined based on the concentrations of competitor peptides needed to prevent 50% binding of the reference peptides as we have described previously (26, 28, 30).

### Creation of MHC Class II/IA^k^ or IE^k^ Dextramers to Determine Antigen-Specificity of T Cells

To enumerate the frequencies of antigen-specific CD4 T cells, we created IA^k^ dextramers for β_1_AR 171-190 and β_1_AR 211–230, and IE^k^ dextramers for β_1_AR 181–200. To create IA^k^ dextramers, the nucleotide sequence for β_1_AR 171–190 (acgcgcgcgcgagcgcgggccctcgtgtgcacagtgtgggccatctcggcgttggtgtcc) and β_1_AR 211–230 (aacgaccccaagtgctgcgatttcgtcaccaacagggcctacgccatcgcctcgtccgtc) was inserted into the IA^k^-β construct that we had described previously (35, 39), whereas IA^k^/RNase 43–56 (control) dextramers were readily available in our laboratory (30, 35, 40). The soluble, IA^k^/β_1_AR 171–190 and IA^k^/β_1_AR 211–230 molecules were expressed in sf9 cells using baculovirus expression system. After purifying through anti-IA^k^-affinity column, the IA^k^/β_1_AR 171–190 and IA^k^/β_1_AR 211–230 monomers were biotinylated and dextramers were derived using streptavidin (SA)/fluorophore-conjugated dextran molecules as described previously (35, 39). IE^k^/β_1_AR 181–200 and IE^k^/MCC 82–103 (control) dextramers were prepared based on peptide-exchange reaction as we have described previously (34).

To stain with dextramers, lymphocytes obtained from lymph nodes and spleens from immunized animals were stimulated with the immunizing peptides namely, β_1_AR_Ac_ 171–190, β_1_AR_Ac_ 181–200, and β_1_AR_Ac_ 211–230 (20 µg/ml) for 2 days and growth medium containing interleukin (IL)-2 (IL-2 medium) was then added. After harvesting viable cells by ficoll density-gradient centrifugation on day 5, cells were rested in the IL-2 medium. During 7–10 days poststimulation, cells were stained with IA^k^-[β_1_AR_Ac_ 171–190 (37°C) or β_1_AR_Ac_ 211–230 (RT), and RNase 43–56, control] or IE^k^-dextramers [β_1_AR_Ac_ 181–200 (RT) and MCC 82–103, control] followed by anti-CD4 and 7-aminoactinomycin-D (7-AAD). After washing, and acquisition by flow cytometry, percent dextramer^+^ (dext^+^) cells were analyzed within the live cells (7-AAD^−^) using Flow Jo software (34, 35, 39).

### Cytokine Secretion

Supernatants were obtained from lymph node cell (LNC) cultures prepared from immunized animals that were stimulated with or without acetylated or non-acetylated, β_1_AR 171–190, β_1_AR 181–200, and β_1_AR 211–230 and RNase 43–56/HEL 46–61 (controls) (50–100 µg/ml) on day 3 poststimulations. Cytokine analysis was performed using beads conjugated with capture and detection Abs with the standard curves being derived from serial dilutions of mouse cytokine standard mixtures, consisting of IL-2, interferon (IFN)-γ, IL-4, IL-6, IL-10, IL-17A, and tumor necrosis factor (TNF)-α as recommended by the manufacturer’s guidelines (BD Biosciences). Briefly, capture bead/cytokine antibody conjugates were first prepared, and the mixtures were added to a tube containing diluted standards or test samples, followed by addition of detection antibodies. After acquiring by flow cytometry, FCAP Array Software was used to analyze the data (BD Biosciences) (26, 30).

### Induction of Myocarditis by Adoptive Transfer of Antigen-Sensitized T Cells

Lymph node cells obtained from animals immunized with individual peptides, β_1_AR_Ac_ 171–190, β_1_AR_Ac_ 181–200, or β_1_AR_Ac_ 211–230 on day 21 postimmunizations were stimulated with the corresponding immunizing peptides (20 µg/ml) for 2 days followed by resting in IL-2 medium. In a separate set of experiments, animals were immunized with a mixture of β_1_AR_Ac_ 171–190, 181–200, and 211–230 peptides and their lymphocytes were stimulated with the immunizing peptides individually (50 µg/ml) for 2 days and then IL-2 medium was added. On day 3, one of the two aliquots of cells were exposed to a mixture of Th1- and Th17-polarizing conditions by adding recombinant mouse IL-12 (20 ng/ml), IL-1β (30 ng/ml), IL-6 (30 ng/ml), IL-23 (50 ng/ml) (all cytokines were procured from BioLegend, San Diego, CA, USA), and human transforming growth factor-β1 (10 ng/ml; eBioscience, San Diego, CA, USA) as reported previously (41–44), and the other was maintained in IL-2 medium alone. Flow cytometrically, we analyzed the percentages of cytokine-producing cells on days 3 and 6 following polarization as we have described previously (28, 31, 40). Viable cells harvested on days 8–10 were administered through i.p., or retro-orbital sinus (4–14 × 10^6^ cells/animal) into naive mice primed with lipopolysaccharide (LPS, 25 µg/mouse i.p., day −4 and day 0). PT was administered i.p., (100 ng/mouse) on days 0 and 2 posttransfer (26, 30, 45). Saline recipients and the LPS/PT-primed naive mice were used as controls. Animals were euthanized on 14–21 days after transfer to collect hearts for histology.

### Detection of Antibodies That React with β_1_AR_Ac_ Peptides

ELISA was used to measure antibodies that react with β_1_AR_Ac_ peptides in the serum samples collected from immunized animals at termination on day 21 as described previously (26). Microtiter polystyrene plates were coated with or without β_1_AR_Ac_ 171–190, β_1_AR_Ac_ 181–200, β_1_AR_Ac_ 191–210, β_1_AR_Ac_ 201–220, and β_1_AR_Ac_ 211–230 or irrelevant control (RNase 43–56) (10 µg/ml) in 1 × coating buffer (eBioscience) and the plates were incubated at 4°C overnight. After washing/blocking, serum samples (1:100) were added in duplicates, and the plates were incubated at 37°C for 1 h followed by addition of HRP-labeled goat anti-mouse immunoglobulins (Igs), IgG1, IgG2a, IgG2b, IgG3, IgM, IgA, and IgE (Southern Biotech, Birmingham, AL, USA) as secondary antibodies. Two hours after incubation at RT, 1 × tetramethylbenzidine solution was added as a substrate (eBioscience), and reactions were stopped using 1 M phosphoric acid. The plates were read at 405 nm using an automated ELISA reader (BioTek instruments, Winooski, VT, USA) to measure OD values (26, 46).

### Statistics

Kruskal–Wallis test was used to compare differences in cytokine production, inflammatory foci and antibodies where more than two groups were involved. When significant differences were noted (*P* < 0.05), analyses were continued with *post hoc* tests *via* Dunn–Sidak multiple test correction. Differences in the incidence of myocarditis between groups were compared using the Fisher’s exact test. Student’s *t*-test was used to determine differences in the T cell proliferative responses, echocardiography parameters, MHC-binding affinities, frequencies of dext^+^ cells, and cytokine producing cells.

## Results and Discussion

In this report, we describe identification of T cell epitopes of β_1_AR in A/J mice that are highly susceptible to lymphocytic myocarditis induced by various cardiac antigens namely, Myhc (28), cardiac troponin I (47), ANT (26), and BCKD kinase (30). Importance of β_1_AR in the cardiac disease has long been investigated in that patients with myocarditis/DCM, in particular, IDCM show β_1_AR-reactive antibodies. Such a reactivity has been proposed to signify a marker of disease-progression and/or to predict prognosis (6, 20). β_1_AR-reactive antibodies localized to EC loop II particularly for β_1_AR 197–222 was also helpful to screen DCM patients in clinical settings (13, 48). Because the sequence of β_1_AR 197–222 is found conserved with 100% identity between humans, mice and rats, and 96.1% with rabbits (Table S1 in Supplementary Material), perhaps, rodent studies were undertaken to determine the pathogenic significance of β_1_AR 197–222 in the development of myocardial disease. Expectedly, rats and rabbits immunized with β_1_AR 197–222 developed cardiac dysfunctions suggestive of DCM, with minimal or no heart infiltrates (21–25). But all these disease-inducing protocols required biweekly or monthly immunizations for a period of 6–18 months. In addition, animals immunized with β_1_AR had agonistic antibodies of IgG isotype that trigger cardiac arrhythmias by activating cAMP/protein kinase A pathway and apoptosis of cardiomyocytes (19). In these conditions, however, relevance of β_1_AR-reactive T cell responses in the causation of cardiac autoimmunity remained uninvestigated. Thus, we made efforts to identify T cell epitopes of β_1_AR and their ability to induce myocarditis in A/J mice.

To identify T cell epitopes, we created an overlapping peptide library that included 46 peptides of 20-mers spanning the entire length of mouse β_1_AR (466 amino acids; Table S2 in Supplementary Material). For initial screening, we made nine pools with four to five peptides in each, and immunized A/J mice using the standard myocarditis-inducing protocol by administering peptide emulsions twice with a week interval as we and others have reported for various cardiac antigens (26, 28, 49). These analyses revealed detection of myocarditis in animals that received peptides from pools namely, IV, VII, VIII, and IX, with a disease incidence of 20–40% and the inflammatory foci were in the range of 2–42 (Table S3 in Supplementary Material). However, by evaluating T cell responses using RNase 43–56 as an irrelevant control, a trend was noted in that animals receiving a pool of peptides starting from β_1_AR 171–190 to β_1_AR 211–230 (pool IV) had good T cell responses up to ~2- to 5-fold that also had diffused inflammatory foci in one animal (Table S3 in Supplementary Material). Similar pattern was noted for one other group (pool VII) with the proliferative responses noted up to two-fold for three peptides (β_1_AR 321–340, β_1_AR 351–370, and β_1_AR 361–380), whereas T cell responses were not significant in the peptide pools, VIII and IX (Table S3 in Supplementary Material). Likewise, significant T cell responses although noted for few peptides in pools, I, II, III, V, and VI, inflammation was absent in the hearts of immunized animals (Table S3 in Supplementary Material). These observations suggest that occurrence of myocarditis might not be due to T cell responses in all the pools.

Since, our intent was to identify T cell epitopes which are capable to induce myocarditis, and the peptides spanning a stretch of 60 aa (β_1_AR 171–230, pool IV; Table S3 in Supplementary Material) that also encompasses β_1_AR 197–222 which was previously reported to be a B cell epitope (13, 48), we focused on all the five peptides from pool IV (Table S4 in Supplementary Material). For comparative analysis, we also randomly chose two other peptides from pool VIII namely; β_1_AR 381–400 and β_1_AR 391–410 that did not show T cell responses, but showed myocarditis (Table S3 in Supplementary Material). In the individual immunization settings, the disease incidence was noted to be 20–40%, but unexpectedly, none of the animals had significant disease, except two peptides that induced mild disease (β_1_AR 181–200 and β_1_AR 211–230) (Table S4 in Supplementary Material).

**Figure 1:**
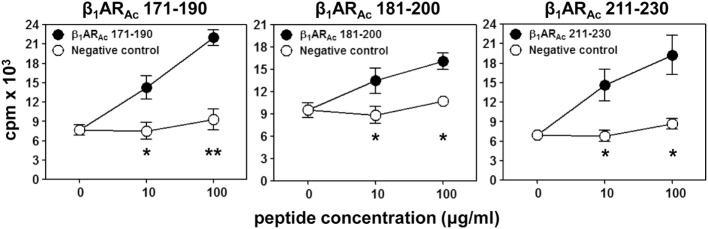
T cell responses induced by β_1_AR_Ac_ peptides. Lymph node cells (LNCs) obtained from animals immunized with β_1_AR_Ac_ 171–190, β_1_AR_Ac_ 181–200, and β_1_AR_Ac_ 211–230 were restimulated with or without the corresponding peptides and RNase 43–56/HEL 46–61 (negative control) for 2 days. After pulsing with ^3^[H]-thymidine for 16 h, proliferative responses were measured as cpm. Mean ± SEM values derived from three to four individual experiments, each involving three to eight mice are shown. *P < 0.05 and **P < 0.01 vs. negative controls.

We and others had previously reported that the peptides modified to include acetyl group at N-terminal end can induce significant myocarditis such as Myhc 614–643 in Balb/c mice (26, 29). It has been proposed that acetylated peptides can be prevented from intracellular degradation leading to their stable binding to MHC molecules (29, 50). Thus, we decided to use the acetylated peptides, and focused only the peptides that were located within the stretch of β_1_AR 171–230 (Table 1). While, some enhancement was noted with the ability of acetylated peptides (β_1_AR_Ac_ 171–190 and β_1_AR_Ac_ 181–200) to induce myocarditis, only 25% of animals had the disease (Table 1). Expectedly, hearts from control groups (naive and CFA/PT) were negative for inflammatory changes. The T cell responses however were consistently noted for β_1_AR_Ac_ 171–190, β_1_AR_Ac_ 181–200, and β_1_AR_Ac_ 211–230 (Figure 1), whereas β_1_AR_Ac_ 191–210 and β_1_AR_Ac_ 201–220 remained as non-T cell activators (Figure S1A in Supplementary Material). The proliferative responses noted in the immunized animals were dose-dependent, and also specific to antigen, since responses to control antigen (RNase 43–56/HEL 46–61) were lacking (Figure 1). We then asked whether T cells primed with a pool of all three peptides that showed T cell responses (β_1_AR_Ac_ 171–190, β_1_AR_Ac_ 181–200, and β_1_AR_Ac_ 211–230) can collectively contribute to myocarditis-induction. These experiments led us to note a significant increase in the disease incidence (63%), but the disease-severity remained unaltered (Table 1, *P* = 0.03).

**Table 1:** Induction of myocarditis by β_1_AR_Ac_ peptides.

Groups	Incidence (%)	Inflammatory foci^a^
Naive	0/6 (0)	0
CFA/PT	0/6 (0)	0
β_1_AR_Ac_ 171–190	3/12 (25)	2.7 ± 1.2
β_1_AR_Ac_ 181–200	3/12 (25)	3.7 ± 1.8
β_1_AR_Ac_ 191–210	0/5 (0)	0
β_1_AR_Ac_ 201–220	1/5 (20)	13
β_1_AR_Ac_ 211–230	2/12 (17)	1.0 ± 0.0
β_1_AR_Ac_ 171–190, 181–200, and 211–230	5/8 (63)*	2.8 ± 1.1

*^a^Represents mean ± SEM values derived from myocarditic animals*.

*P < 0.05 vs. controls.

**Figure 2:**
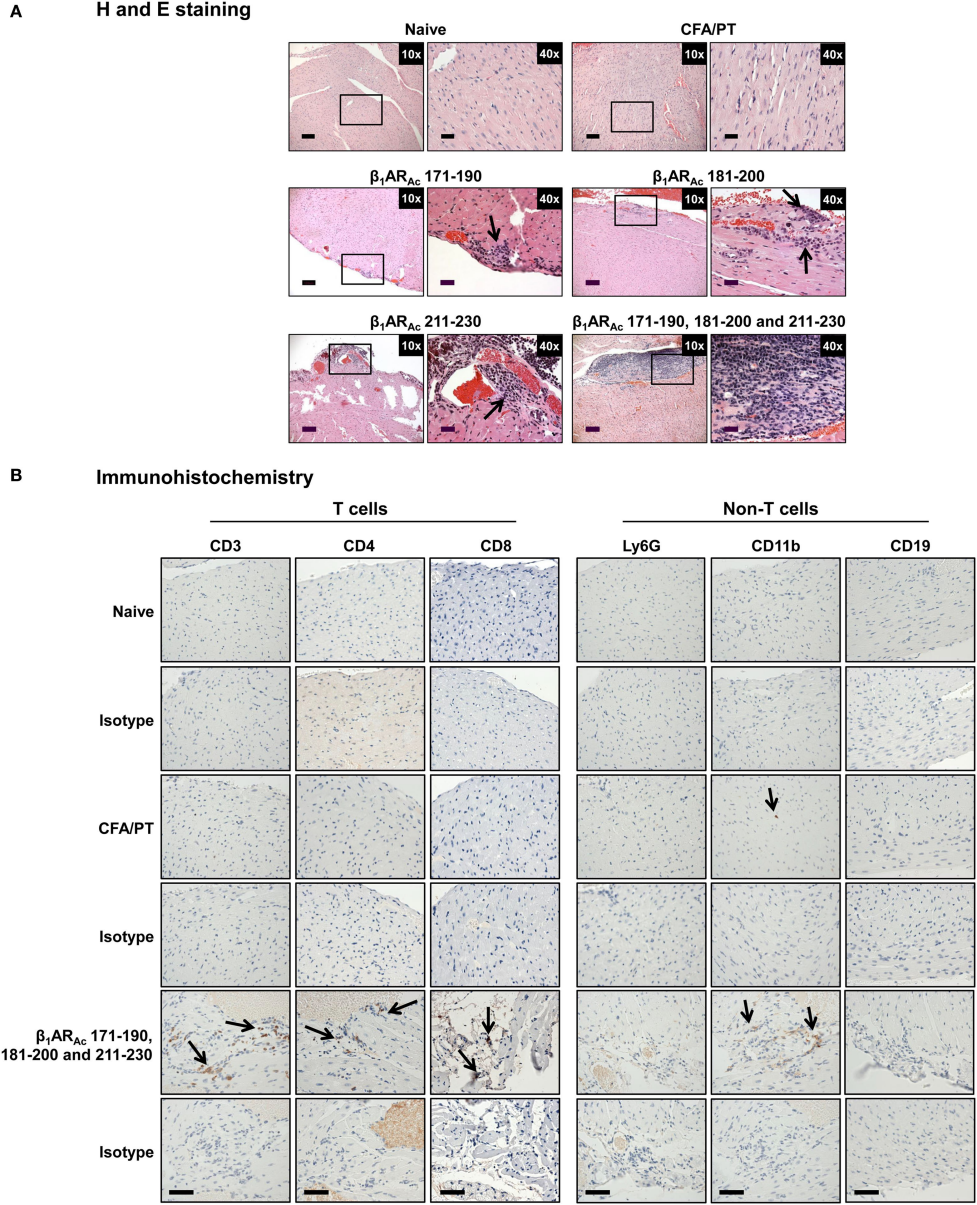
Histological evaluation of hearts from animals immunized with β_1_AR_Ac_ peptides. **(A)** Hematoxylin and eosin (H&E) staining. Groups of mice were immunized with or without indicated peptides in complete Freund’s adjuvant (CFA) twice with an interval of 7 days. At termination on day 21, hearts were collected to determine inflammatory changes by H&E staining analysis. Mild myocarditis as indicated by infiltrates containing mononuclear cells (MNCs) were noted in all groups. Boxed areas are shown at higher magnifications to the right in each panel. Arrows, inflammatory foci. Scale bar: 120 µm (overview images, 10×), 30 µm (magnifications, 40×). **(B)** Immunohistochemistry. Heart sections obtained from animals immunized with a mixture of β_1_AR_Ac_ 171–190, 181–200, and 211–230 were evaluated for the presence of T cells (CD3, CD4, and CD8) and non-T cells (Ly6G^+^ neutrophils, CD11b^+^ macrophages and CD19^+^ B cells) using antibodies for each marker or their isotype controls. After washing and addition of horseradish peroxidase (HRP)-conjugated secondary antibodies, cells positive for each marker were identified as shown with arrows. Representative sections from groups involving five to eight mice are shown. Naive and CFA/PT, controls; and scale bar, 60 µm.

Histologically, in contrast to normal heart sections, inflammatory foci consisting of mononuclear cells (MNCs) involving pericardium, myocardium and endocardium were detected in animals immunized with β_1_AR_Ac_ 171–190, β_1_AR_Ac_ 181–200, or β_1_AR_Ac_ 211–230 individually or as a mixture, but necrosis or fibrosis were absent (Figure 2A). Consistent with these observations, echocardiographic analysis also revealed mild cardiac abnormalities as indicated by trends with increased heart rate relative to control animals (384.0 ± 10.10 vs. 337.67 ± 20.98 beats/min; *P* = 0.08, Table S5 in Supplementary Material). Immunized animals showed decreased LVID index at end-systole (0.26 ± 0.01 vs. 0.33 ± 0.01 mm; *P* = 0.047) and end-systolic volume (17.50 ± 2.89 vs. 33.33 ± 8.33 µl; *P* = 0.09, Table S5 in Supplementary Material) associated with an increase in left ventricular systolic function reported as ejection fraction and fractional shortening. The immunized mice showed the absence of hypertrophy and dilation as indicated by the lack of significant increase in the diastolic septal/posterior wall thickness indices and end-diastolic volume. However, the RWT of 0.43 in the immunized mice (normal RWT = 0.32–0.42) identifies concentric remodeling without hypertrophy whereas RWT of 0.39 identifies a normal morphology as seen in the naive mice (51). No differences with body weight (19.20 ± 0.32 vs. 18.23 ± 0.44 g), heart weight (87.25 ± 1.11 vs. 84.00 ± 1.73 mg) or heart/body weight ratio (4.55 ± 0.03 vs. 4.61 ± 0.07 mg/g) were observed between immunized and control groups (Figure S2 in Supplementary Material). Previous reports indicate that rats immunized with β_1_AR 197–222 for a prolonged period showed early cardiomyopathic changes at 1 year, but with no necrosis or fibrosis including changes in heart to body weight ratios (21). Likewise, cardiac abnormalities could be detected in Balb/c mice only at 25 weeks but not at 14 weeks postimmunization (22). These observations support the notion that β_1_AR-induced cardiomyopathy may be a slow progressive disease.

Nonetheless, we further characterized the immune cells by IHC and the analysis revealed that heart sections from animals immunized with a mixture of three peptides as indicated above had CD3^+^ (136 ± 9 cells/mm^2^), CD4^+^ (92 ± 1 cells/mm^2^), CD8^+^ (66 ± 21 cells/mm^2^) T cells, and CD11b^+^ (65 ± 13 cells/mm^2^) macrophages, whereas Ly6G^+^ neutrophils, and CD19^+^ B cells were absent (Figure 2B: bottom panel). Expectedly, sections from control groups were negative for all the markers tested except rare CD11b^+^ cells. While, detection of T cells, mainly CD4 T cells and macrophages suggest the features of delayed-type hypersensitivity (DTH) reaction as noted with other myocarditis models (26, 28–30), detection of CD8 T cells was not expected although they can form a component of DTH reaction. This led us to test a possibility that both CD4 and CD8 T cells may have a role in myocarditis-induction. We sorted both subsets of T cells by magnetic separation from animals immunized with β_1_AR_Ac_ 171–190, β_1_AR_Ac_ 181–200, or β_1_AR_Ac_ 211–230 to a purity of ~95% (Figure S3A in Supplementary Material), and tested their ability to respond to antigens in a proliferation assay. As shown in Figure S3B in Supplementary Material, both CD4 and CD8 T cells responded to the corresponding immunizing peptides dose-dependently, and also antigen-specifically, since responses to HEL 46–61 were lacking. While, these data suggest that the disease induced with β_1_AR_Ac_ 171–190, β_1_AR_Ac_ 181–200, and β_1_AR_Ac_ 211–230 might have involved the mediation of both CD4 and CD8 T cells, existence of three CD8 T cell epitopes in as many CD4 T cell epitopes for a single autoantigen is unusual. But, we and others have previously reported that CD4 T cell epitopes of 19–21-mers can act as common epitopes for both CD4 and CD8 T cells as shown with Myhc 334–352 (31), myelin oligodendrocyte glycoprotein 35–55 (52), and interphotoreceptor retinoid-binding protein 1–20 (53). In the present study however, we did not investigate localization of CD8 T cell epitopes in the β_1_AR peptides, and such an identity may then create opportunities to determine how CD8 T cells independently or together with CD4 T cells can induce myocarditis, since their effector functions are distinct (CD8 cytotoxic vs. CD4 Th).

**Figure 3:**
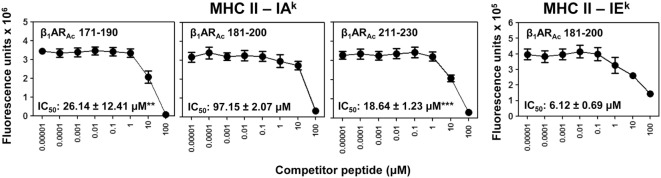
Determination of major histocompatibility complex (MHC) class II-binding affinities of β_1_AR_Ac_ peptides. Mixtures containing thrombin-cleaved IA^k^ and IE^k^ soluble monomers (0.35 µg), competitor peptides—β_1_AR_Ac_ 171–190, β_1_AR_Ac_ 181–200, and β_1_AR_Ac_ 211–230 (0.00001–100 µM)—and biotinylated hen egg lysozyme (HEL) 46–61 (reference for IA^k^) and moth cytochrome *c* (MCC) 82–103 (reference for IE^k^) (1 µM) were prepared and added to fluorescence plates coated with anti-IA^k^ or IE^k^ in duplicates as described in the methods. After washing, and addition of europium-labeled streptavidin (SA) and dissociation-enhanced lanthanide fluoroimmunoassay (DELFIA) enhancer, fluorescence intensities were measured at excitation/emission wavelengths of 340/615 nm to calculate the IC_50_ values. Mean ± SEM values from three individual experiments with two replicates in each are shown. **P < 0.01 and ***P < 0.001 vs. IC_50_ of IA^k^/β_1_AR_Ac_ 181–200.

**Figure 4:**
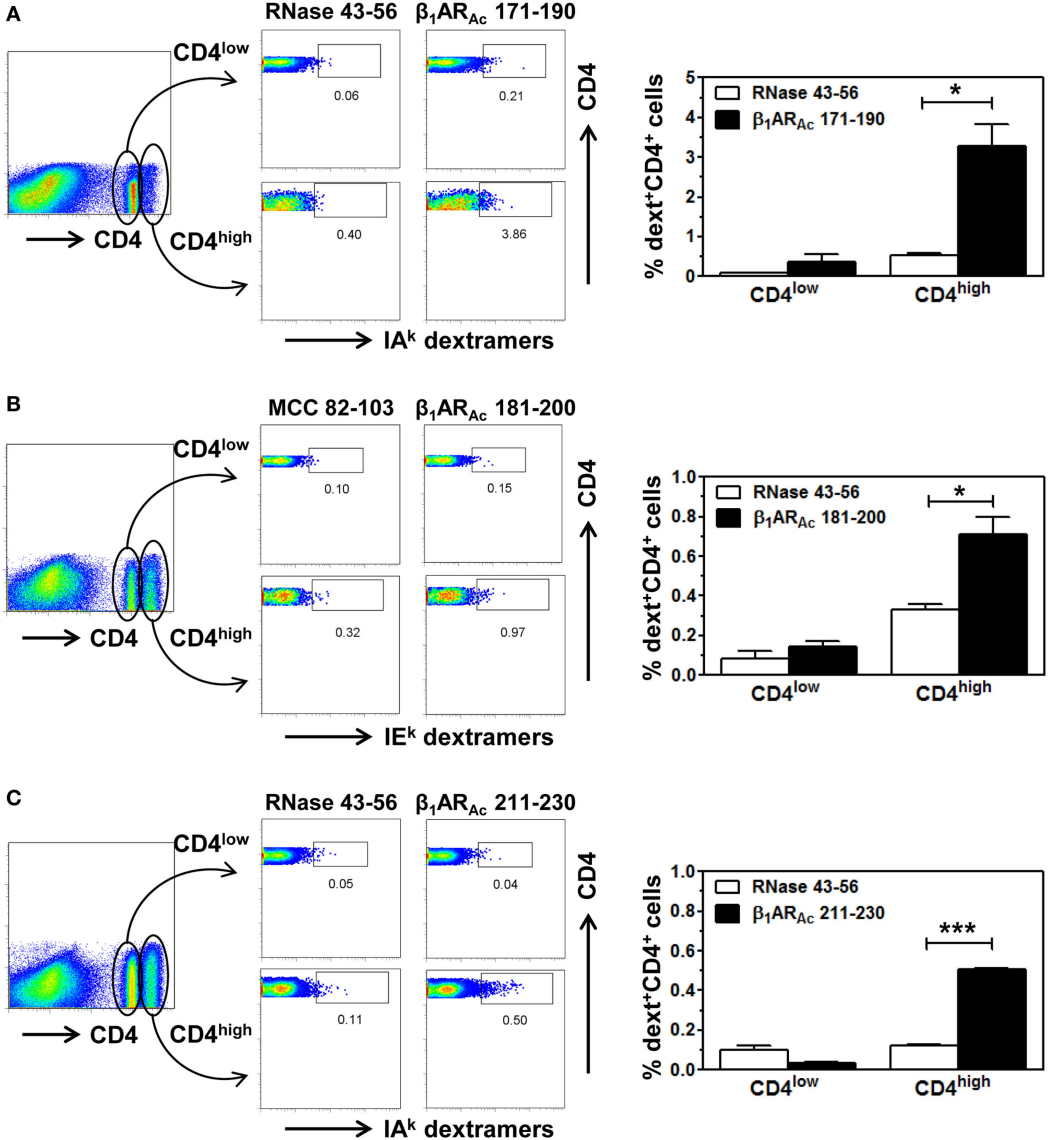
Antigen-specificity of T cell responses induced by β_1_AR_Ac_ peptides. Lymph node cells (LNCs) or splenocytes prepared from animals immunized with β_1_AR_Ac_ 171–190, β_1_AR_Ac_ 181–200, and β_1_AR_Ac_ 211–230 were restimulated with the corresponding peptides for 2 days and cells were rested in interleukin (IL)-2 medium. Cells harvested on days 7–10 poststimulation were stained with three sets of dextramers namely, IA^k^ dextramers (β_1_AR_Ac_ 171–190 and RNase 43–56 as control), panel **(A)**; IE^k^ dextramers [β_1_AR_Ac_ 181–200 and moth cytochrome *c* (MCC) 82–103 as control], panel **(B)**; and IA^k^ dextramers (β_1_AR_Ac_ 211–230 with RNase 43–56 as control), panel **(C)**; followed by staining with anti-CD4 and 7-aminoactinomycin-D (7-AAD). Cells were acquired by flow cytometry, and the dext^+^ cells were analyzed corresponding to CD4^low^ or CD4^high^ populations using Flow Jo software (bar graphs on the right side in each panel). Representative flow cytometric plots are shown from two to three individual experiments, with two to five mice per group. *P < 0.05 and ***P < 0.001 vs. control dextramers.

Further, we made efforts to characterize CD4 T cell responses as to their antigen-specificity. First, we determined MHC class II-binding affinities of β_1_AR_Ac_ peptides using soluble MHC class-II/IA^k^ and IE^k^ monomers (26, 28, 30). These analyses revealed two of the three peptides that induced T cell responses (Figure 1) namely, β_1_AR_Ac_ 171–190 and β_1_AR_Ac_ 211–230 could bind IA^k^ molecules as indicated by their respective IC_50_ values, 26.14 ± 12.41 and 18.64 ± 1.23 µM, whereas the binding affinity of β_1_AR_Ac_ 181–200 was low (97.15 ± 2.07 µM) (Figure 3, left panel; *P* < 0.01). Of note, in a comparative analysis, another peptide, β_1_AR_Ac_ 201–220 that did not induce T cell response failed to bind IA^k^ molecule (Figure S1B in Supplementary Material), and it was not chosen for further characterization. Since, β_1_AR_Ac_ 181–200, a T cell activator was found to be a poor binder of IA^k^, we generated IE^k^ monomers to determine its ability to bind IE^k^ molecule, since A/J mice express both IA^k^ and IE^k^ molecules (33, 34). By using IE^k^ monomers, and MCC 82–103 as a reference peptide, we determined that β_1_AR_Ac_ 181–200 to be a good binder of IE^k^ molecule as indicated by the IC_50_ value 6.12 ± 0.69 µM (Figure 3, right panel). Based on this information, we decided to create two sets of dextramers: IA^k^ dextramers for β_1_AR_Ac_ 171–190 and β_1_AR_Ac_ 211–230 with RNase 43–56 as control, and IE^k^ dextramers for β_1_AR_Ac_ 181–200 and MCC 82–103 (control). Using these reagents, we tested the antigen-specificity of T cell responses in animals immunized with β_1_AR_Ac_ 171–190, β_1_AR_Ac_ 181–200, and β_1_AR_Ac_ 211–230, respectively. CD4 T cells from cultures stimulated with the respective peptides were found to bind dextramers antigen-specifically, whereas the staining intensity obtained with the control dextramers was low as determined by flow cytometry (Figure 4). As we have reported previously for various self- and foreign-antigens (28, 34, 35, 54), we noted that the dextramers for all the three T cell epitopes of β_1_AR were also preferentially found to bind activated CD4^high^ than CD4^low^ subset. The percent dext^+^ CD4^high^ T cells were: β_1_AR_Ac_ 171–190 (3.27 ± 0.56%) vs. RNase 43–56 (0.52 ± 0.08%) (*P* = 0.049, Figure 4A), β_1_AR_Ac_ 181–200 (0.71 ± 0.09%) vs. MCC 82–103 (0.33 ± 0.03%) (*P* = 0.028, Figure 4B), and β_1_AR_Ac_ 211–230 (0.51 ± 0.01%) vs. RNase 43–56 (0.12 ± 0.01%) (*P* = 0.0003, Figure 4C). The data point to a possibility that the antigen-sensitized CD4 T cells might be the mediators of disease.

**Figure 5:**
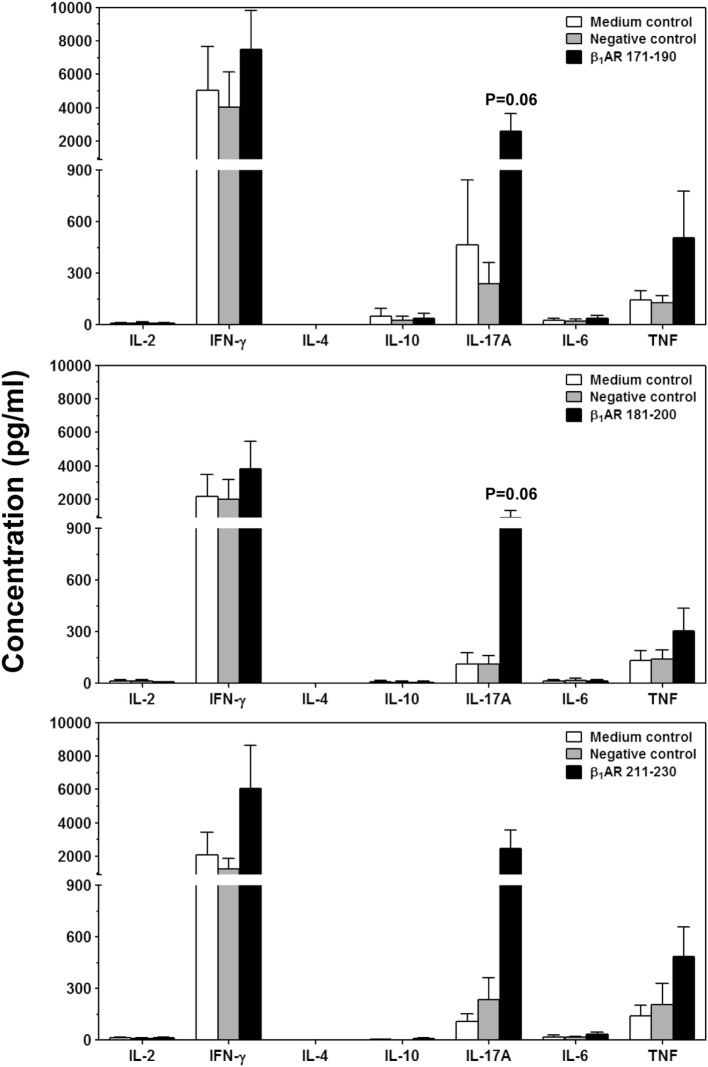
Cytokine responses induced by β_1_AR_Ac_ peptides. Lymph node cells (LNCs) were prepared from animals immunized with acetylated or non-acetylated β_1_AR 171–190, β_1_AR 181–200, or β_1_AR 211–230, and after restimulating with or without the immunizing peptides or RNase 43–56/HEL 46–61 (negative controls), culture supernatants were harvested on day 3. Samples were analyzed for indicated cytokines by bead array analysis as described in the methods section. Each bar represents mean ± SEM values derived from four to five individual experiments, with three to eight mice per group.

Next, we sought to determine the pathogenic potential of T cells sensitized with β_1_AR 171–190, β_1_AR 181–200, or β_1_AR 211–230 by analyzing their ability to produce various inflammatory cytokines (Figure 5). Supernatants from cultures stimulated with or without specific or control (RNase 43–56/HEL 46–61) antigens revealed the presence of all cytokines, except IL-4. Data revealed no striking differences for any of the cytokines except that IL-17A was tended to be elevated in β_1_AR 171–190 and β_1_AR 181–200 cultures (*P* = 0.06, Figure 5). Conversely, production of IFN-γ, another key cytokine was not altered including other proinflammatory (IL-6 and TNF-α) or anti-inflammatory (IL-4 and IL-10) cytokines. Accumulated literature suggests that IFN-γ can act as a disease-protective or disease-inducing cytokine in various myocarditis models, and such differential effects may be antigen-dependent (55). Likewise, IL-17A appears not to be required for initiating the disease, but plays a critical role for progression of myocarditis (56). We have also seen that cardiac-reactive T cells produce mostly IFN-γ (30) and/or IL-17A in addition to IL-6 and TNF-α in A/J mice (26, 28). Since, IFN-γ-production was found to be unaltered with β_1_AR peptides, production of IL-17A alone might be insufficient to initiate myocarditis which is consistent with the observations made in Myhc 614–629-induced myocarditis (56). TNF-α and IL-6, being inflammatory cytokines may have a role in myocarditis (57, 58), but their amounts were low in cultures stimulated with β_1_AR peptides to predict their impact. The finding that IL-4 or IL-10 were detected in insignificant amounts in all the cultures, suggests that the mild nature of disease in the immunized animals appear not due to immune deviation occurring toward Th2 phenotype.

**Figure 6:**
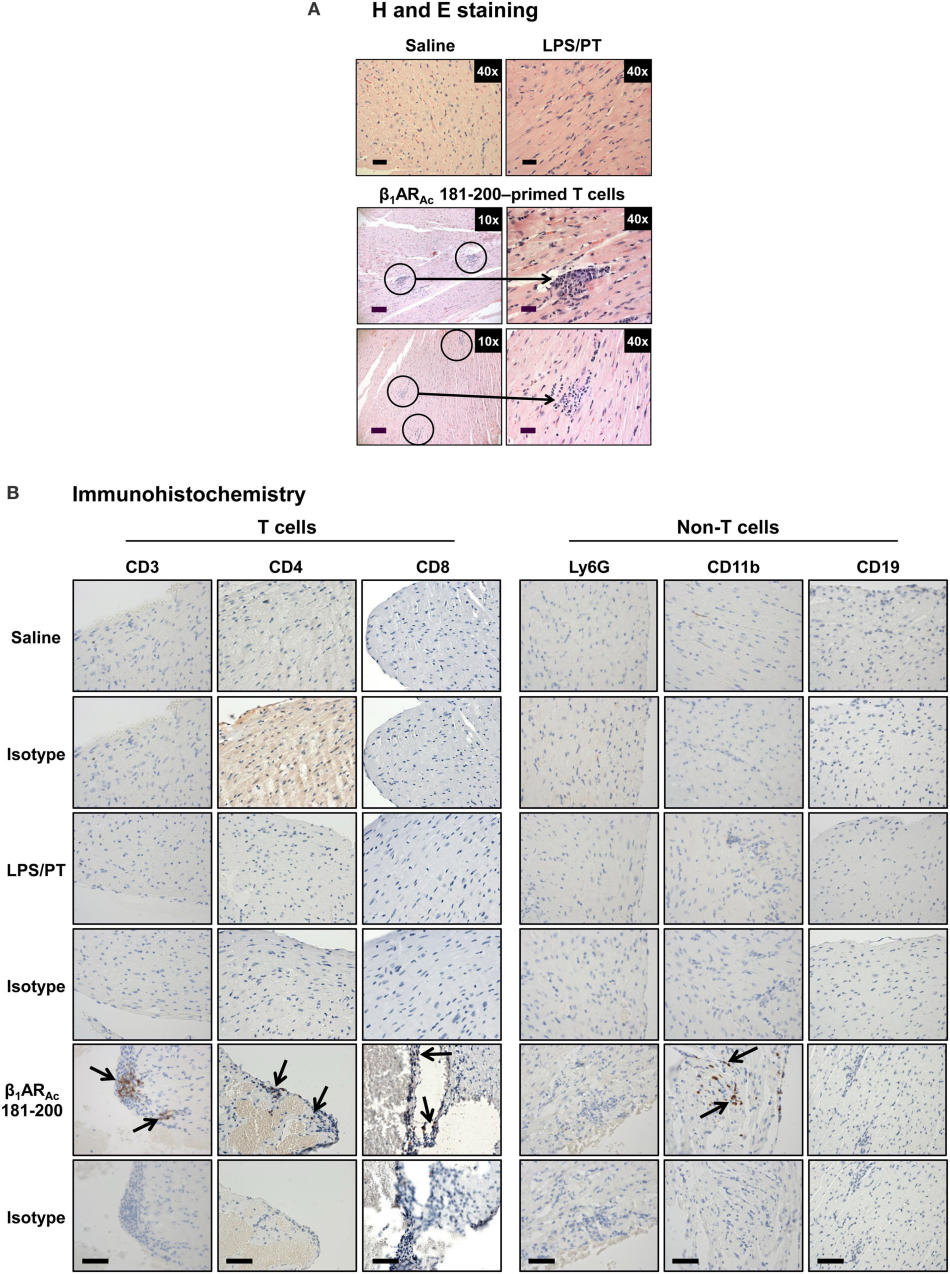
Evaluation of hearts for inflammatory changes in naive recipients of T cells sensitized with β_1_AR_Ac_ 181–200. **(A)** Hematoxylin and eosin (H&E) staining. Groups of mice were immunized with a mixture of β_1_AR_Ac_ 171–190, β_1_AR_Ac_ 181–200, and β_1_AR_Ac_ 211–230 and after 14–21 days, lymph node cells (LNCs) were restimulated with the corresponding peptides for 2 days. After exposing to T helper (Th)1- and Th17-polarizing conditions, cells harvested on day 7 postpolarization and administered into naive mice primed with lipopolysaccharide (LPS). Representative normal heart sections in saline and LPS/PT controls are shown in the top panel. The bottom panel represents recipients of β_1_AR_Ac_ 181–200-sensitized/polarized T cells. Circled areas are shown at higher magnifications to the right in each panel (arrows, inflammatory foci). Scale bar: 120 µm (overview images, 10×), 30 µm (magnifications, 40×). *n* = 4 mice per group. **(B)** Immunohistochemistry. Heart sections were examined for T cells and non-T cells using the antibodies for indicated markers. Detection of cells positive for each marker (arrows) was analyzed by adding HRP-conjugated secondary antibodies as described in the methods section. Representative sections from four animals in each group are shown. Scale bar, 60 µm.

To investigate whether β_1_AR-reactive cells producing differential amounts of Th1 and Th17 cytokines could determine the disease outcome, we performed adoptive transfer experiments using antigen-primed T cells treated with or without a cocktail of cytokines that polarize toward Th1 and Th17 responses (41–44). As shown in Figure S4 in Supplementary Material, we noted increase in the frequencies of cells expressing Th1 (IL-2 and IFN-γ) and Th17 (IL-17A and IL-22) including Th2 (IL-4 and IL-10) cytokines as reported by others (41–44, 59, 60). However, comparative analysis revealed Th1-producing cells to be significantly enhanced in β_1_AR 181–200 cultures as opposed to Th2 cytokine-producing cells in β_1_AR_Ac_ 171–190 and β_1_AR_Ac_ 211–230 cultures but with no significant increase in the Th17 subset in any of the groups. Table 2 shows that the naive animals receiving unpolarized lymphocytes specific to β_1_AR_Ac_ 171–190 and β_1_AR_Ac_ 211–230 had no inflammatory foci in their hearts, where as one animal receiving β_1_AR_Ac_ 181–200-primed lymphocytes had heart lesions. When the cells were exposed to the polarizing conditions as described above, it was clear that the naive animals receiving β_1_AR_Ac_ 181–200-primed lymphocytes had severe myocarditis (25.8 ± 7.4 foci; Table 2; Figure 6A; Figure S5 in Supplementary material). Under similar conditions, β_1_AR_Ac_ 171–190-and β_1_AR_Ac_ 211–230-responsive lymphocytes did not result in disease, which may be due to their enhanced ability to produce Th2 cytokines (Table 2; Figure S4 in Supplementary Material). Expectedly, heart sections from control groups (saline and LPS/PT) also were negative for inflammatory changes (Table 2; Figure 6A). Furthermore, by IHC analysis, we noted detection of CD3^+^, CD4^+^ and CD8^+^ T cells and also CD11b^+^ macrophages in heart sections from animals that received β_1_AR_Ac_ 181–200-primed/polarized lymphocytes (Figure 6B). These findings may reinforce the notion that the disease induced with β_1_AR_Ac_ peptides represents a T cell-mediated, disease as we have demonstrated with other antigens namely, Myhc, ANT, and BCKD kinase (26, 28, 30). While, cytokine-polarized experiments provided explanations as to why the disease-severity was mild in animals that received CFA/β_1_AR_Ac_ peptide emulsions by active immunizations, occurrence of disease with only β_1_AR_Ac_ 181–200-sensitized T cells, but not for two other peptides (β_1_AR_Ac_ 171–190 and β_1_AR_Ac_ 211–230) in adoptive transfer experiments may indicate that both Th1 and Th17 cytokines are critical for disease induction. Alternatively, it is possible that cytokines other than Th1 and Th17 subsets may have a role in the disease-mediation with β_1_AR_Ac_ 181–200-sensitized T cells because comparison of frequencies of cytokine-producing cells between groups did not reveal striking differences for any of the cytokines tested.

**Table 2:** Myocarditis induced by T cells sensitized with β_1_AR_Ac_ peptides in naive A/J mice.

Groups	Incidence (%)	Inflammatory foci^a^
Saline	0/4 (0)	0
LPS/PT	0/4 (0)	0
**Unpolarized cells**		
β_1_AR_Ac_ 171–190	0/4 (0)	0
β_1_AR_Ac_ 181–200	1/4 (25)	6
β_1_AR_Ac_ 211–230	0/4 (0)	0
**Th1- and Th17-polarized cells**		
β_1_AR_Ac_ 171–190	0/4 (0)	0
β_1_AR_Ac_ 181–200	4/4 (100)*	25.8 ± 7.4**
β_1_AR_Ac_ 211–230	1/4 (25)	1

*^a^Represents mean ± SEM values derived from myocarditic animals*.

*P < 0.05 vs. control groups.

**P < 0.01 vs. control groups.

**Figure 7:**
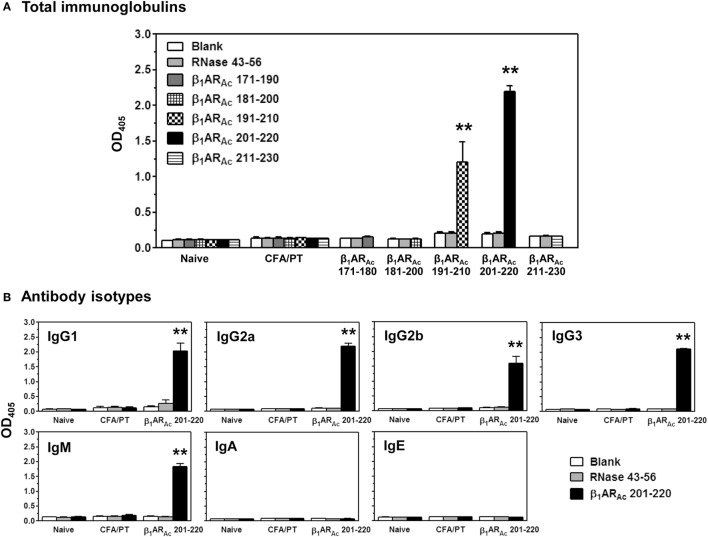
Antibody responses induced by β_1_AR_Ac_ peptides. Groups of mice were immunized with β_1_AR_Ac_ 171–190, β_1_AR_Ac_ 181–200, β_1_AR_Ac_ 191–210, β_1_AR_Ac_ 201–220, or β_1_AR_Ac_ 211–230 and after 21 days, serum was collected. Samples with dilutions of 1:100 were added to low-binding plates coated previously with or without the above peptides or RNase 43–56 (control). After adding horseradish peroxidase (HRP)-conjugated, goat anti-mouse detection antibodies for Igs and different isotypes, plates were read at 405 nm to measure the OD values. Each bar represents mean ± SEM values derived from five animals per group. **P < 0.01 vs. controls. **(A)** Total immunoglobulins and **(B)** antibody isotypes.

Finally, we evaluated antibody responses for a panel of β_1_AR_Ac_ peptides. These include peptides that induced both T cell responses and disease (β_1_AR_Ac_ 171–190, β_1_AR_Ac_ 181–200, and β_1_AR_Ac_ 211–230), and those that did not induce T cell responses or disease (β_1_AR_Ac_ 191–210 and β_1_AR_Ac_ 201–220). Serum samples collected from immunized animals were tested for their reactivity to the immunizing peptides as indicated above or an irrelevant control, RNase 43–56. The analyses revealed detection of total Igs specific to β_1_AR_Ac_ peptides, since such a reactivity was found lacking for RNase 43–56 (Figure 7A). However, comparison of antibody responses between different peptides revealed antibody response to β_1_AR_Ac_ 201–220 and to some degree, β_1_AR_Ac_ 191–210 (Figure 7A). By further characterizing various isotypes, we noted that the antibody response induced with β_1_AR_Ac_ 201–220 involved the production of all IgG isotypes (IgG1, IgG2a, IgG2b, IgG3) including IgM, except IgA and IgE (Figure 7B). By being a non-T cell activator (Figure S1A in Supplementary Material), production of various isotypes of Abs specific to β_1_AR_Ac_ 201–220 may mean that the antigen-specific B cells might have received cytokines from non-T cell sources for isotype switching to occur. One potential source is the adjuvant (CFA) that can promote isotype switching through the secretion of Type I IFNs from dendritic cells as shown previously (61). Furthermore, it is to be noted that β_1_AR_Ac_ 201–220 encompasses 20 amino acids of the previously reported B cell epitope, β_1_AR 197–222 that was shown to induce antibodies in various rodent species (21, 24, 25). Our data also supports this observation. Detection of antibodies to β_1_AR_Ac_ 191–210 may be due to the presence of overlapping sequence between β_1_AR_Ac_ 191–210 and β_1_AR_Ac_ 201–220. However, the observation that β_1_AR_Ac_ 201–220 failed to induce myocarditis in our model may not necessarily mean that the β_1_AR_Ac_ 201–220-induced antibodies are not pathogenic. The reason for this discrepancy is that animals needed to be repeatedly immunized with β_1_AR_Ac_ 197–222 for a period of 12 months in rats and rabbits, and mice were also to be immunized with six doses of CFA/peptide emulsions (21–25). We did not investigate whether A/J mice immunized for such a prolonged period can develop myocarditis, since we limited our focus to the extent of identifying T cell epitopes, and their ability to induce myocarditis in a standard disease-inducing protocol.

In summary, we demonstrate existence of multiple immunodominant epitopes that are unique to T cells or B cells, but not both. Use of a single motif, β_1_AR 197–222 as a B cell epitope to determine the mechanisms of antibody-mediated myocardial disease in several rodent species was perhaps on the idea that the epitope was found conserved (Table S1 in Supplementary Material). Along the same lines, comparison of sequences between humans, mice, rats and rabbits revealed 100% identity for all T cell epitopes that we have described namely, β_1_AR 181–200, and β_1_AR 211–230 with only one residue being different for β_1_AR 171–190 between humans and mice (Table S6 in Supplementary Material), suggest that these epitopes may be relevant to evaluate T cell responses in humans. In addition, we noted that splenocytes from naive animals responded to two out of three T cell epitopes namely, β_1_AR_Ac_ 171–190, and β_1_AR_Ac_ 181–200 (Figure S6 in Supplementary Material) indicating that the naive repertoire can contain a proportion of β_1_AR-reactive T cells that might have responded in response to peptide immunizations. Reports suggest that β_1_AR proteins are differentially expressed in the thymus with a predominant form being β_2_AR (62). It is possible that the developing thymocytes may escape from thymic tolerance mechanisms due to low β_1_AR-expression in the thymus leading to their emigration to the periphery. Thus, we speculate that the preexisting β_1_AR-reactive T cells can be activated under conditions of self-tolerance to be broken by environmental triggers. Of note, cross-reactive immune responses have been noted between *Trypanosoma cruzi* and β_1_AR and it might be interesting to determine whether such responses are directed against the T cell epitopes in A/J mice which are also highly susceptible to *T. cruzi* infection (63, 64). Additionally, we have demonstrated that the β_1_AR-reactive T cells producing insufficient amounts of IFN-γ can become pathogenic under Th1-polarized conditions. Thus, we could envision a scenario that IL-12 produced by the innate immune cells such as macrophages and dendritic cells in response to intracellular pathogens like *T. cruzi* can possibly trigger IFN-γ-producing β_1_AR-reactive T cells to become pathogenic. Such a possibility exists for three reasons: (a) β_1_AR is preferentially expressed in the heart; (b) evaluation of non-cardiac tissues such as brain, liver, lung, and kidney in animals immunized with β_1_AR peptides did not reveal any significant inflammatory changes relative to control groups (Table S7 in Supplementary Material); and (c) all the three T cell epitopes are unique to β_1_AR because they share only 40–65% similarity with β_2_AR and β_3_AR (Table S8 in Supplementary Material). Thus, the pathogenic β_1_AR-reactive T cells are expected to mediate damage preferentially in the heart.

## Ethics Statement

All experiments with mice were performed in accordance with the regulations of and with the approval of the Institutional Animal Care and Use Committee, University of Nebraska-Lincoln, Lincoln, NE, USA (protocol #: 1398).

## Author Contributions

Conceived and designed the experiments: RHB, CM, AG, and JR. Performed the experiments: RHB, BK, CM, RAR, MZA, and JLS. Analyzed the data: RHB, BK, AG, J-JR, and DS. Wrote the article: RHB and JR.

## Conflict of Interest Statement

The authors declare that the research was conducted in the absence of any commercial or financial relationships that could be construed as a potential conflict of interest.

## Abbreviations

DCM, dilated cardiomyopathy; β_1_AR, β1-adrenergic receptor; β_1_AR_Ac_, N-terminal acetylated β1-adrenergic receptor; IDCM, idiopathic dilated cardiomyopathy; ANT, adenine nucleotide translocator 1; BCKD, branched chain alpha-ketoacid dehydrogenase; Myhc, cardiac myosin heavy chain-α; EC, extracellular; cAMP, cyclic adenosine monophosphate; MHC, major histocompatibility complex; Th, T helper; RNase, bovine ribonuclease; HEL, hen egg lysozyme; MCC, moth cytochrome *c*; CFA, complete Freund’s adjuvant; PT, pertussis toxin; H&E, hematoxylin and eosin; IHC, immunohistochemistry; Abs, antibodies; RT, room temperature; HRP, horseradish peroxidase; LV, left ventricle; LVID, left ventricular internal diameter; RWT, relative wall thickness; BSA, body surface area; ^3^[H]-thymidine, tritiated-thymidine; cpm, counts per minute; SA, streptavidin; DELFIA, dissociation-enhanced lanthanide fluoroimmunoassay; LNCs, lymph node cells; 7-AAD, 7-aminoactinomycin-D; dext^+^, dextramer^+^; IL, interleukin; IFN, interferon; TNF, tumor necrosis factor; LPS, lipopolysaccharide; MNCs, mononuclear cells; *T. cruzi, Trypanosoma cruzi*.
