# Supplementary material for: β1-Adrenergic Receptor Contains Multiple IAk and IEk Binding Epitopes That Induce T Cell Responses with Varying Degrees of Autoimmune Myocarditis in A/J Mice
Source: Front Immunol. 2017 Nov 20;8:1567. doi: 10.3389/fimmu.2017.01567 (PMC5701947; doi:10.3389/fimmu.2017.01567)
Supplement: Supplementary file 1 [file Table_1.PDF]

Table S1. Comparison of β_1AR 197-222-sequences among different species.

Species	Location	Sequence	Identity (%)
Human	β_1AR 197-222	HWWRAESDEARRCYNDPKCCDFVTNR	
Mouse	β_1AR 197-222	HWWRAESDEARRCYNDPKCCDFVTNR	100
Rat	β_1AR 197-222	HWWRAESDEARRCYNDPKCCDFVTNR	100
Rabbit	β_1AR 244-269	HWWRAE**G**DEARRCYNDPKCCDFVTNR	96.1

Non-identical residue is bolded and underlined
