# Supplementary material for: β1-Adrenergic Receptor Contains Multiple IAk and IEk Binding Epitopes That Induce T Cell Responses with Varying Degrees of Autoimmune Myocarditis in A/J Mice
Source: Front Immunol. 2017 Nov 20;8:1567. doi: 10.3389/fimmu.2017.01567 (PMC5701947; doi:10.3389/fimmu.2017.01567)
Supplement: Supplementary file 2 [file Table_2.PDF]

Table S2. The list of overlapping peptides of β_1AR used in the study.

Peptides	Sequence
β_1AR 1-20	MGAGALALGASEPCNLSSAA
β_1AR 11-30	SEPCNLSSAAPLPDGAATAA
β_1AR 21-40	PLPDGAATAARLLVLASPPA
β_1AR 31-50	RLLVLASPPASLLPPASEGS
β_1AR 41-60	SLLPPASEGSAPLSQQWTAG
β_1AR 51-70	APLSQQWTAGMGLLLALIVL
β_1AR 61-80†	MGLLLALIVLLIVVGNVLVI
β_1AR 71-90	LIVVGNVLVIVAIAKTPRLQ
β_1AR 81-100	VAIAKTPRLQTLTNLFIMSL
β_1AR 91-110	TLTNLFIMSLASADLVMGLL
β_1AR 101-120	ASADLVMGLLVVPFGATIVV
β_1AR 111-130	VVPFGATIVVWGRWEYGSFF
β_1AR 121-140	WGRWEYGSFFCELWTSVDVL
β_1AR 131-150	CELWTSVDVLCVTASIETLC
β_1AR 141-160†	CVTASIETLCVIALDRYLAI
β_1AR 151-170	VIALDRYLAITSPFRYQSLL
β_1AR 161-180	TSPFRYQSLLTRARARALVC
β_1AR 171-190	TRARARALVCTVWAISALVS
β_1AR 181-200	TVWAISALVSFLPILMHWWR
β_1AR 191-210	FLPILMHWWRAESDEARRCY
β_1AR 201-220	AESDEARRCYNDPKCCDFVT
β_1AR 211-230	NDPKCCDFVTNRAYAIASSV
β_1AR 221-240	NRAYAIASSVVSFYVPLCIM
β_1AR 231-250	VSFYVPLCIMAFVYLRVFRE
β_1AR 241-260	AFVYLRVFREAQKQVKKIDS
β_1AR 251-270	AQKQVKKIDSCERRFLGGPA
β_1AR 261-280	CERRFLGGPARPPSPEPSPS
β_1AR 271-290	RPPSPEPSPSPGPPRPADSL
β_1AR 281-300	PGPPRPADSLANGRSSKRRP
β_1AR 291-310	ANGRSSKRRPSRLVALREQK
β_1AR 301-320	SRLVALREQKALKTLGIIMG
β_1AR 311-330	ALKTLGIIMGVFTLCWLPFF
β_1AR 321-340	VFTLCWLPFFLANVVKAFHR
β_1AR 331-350	LANVVKAFHRDLVPDRLFVF
β_1AR 341-360	DLVPDRLFVFFNWLGYANSA
β_1AR 351-370	FNWLGYANSAFNPIIYCRSP
β_1AR 361-380	FNPIIYCRSPDFRKAFQRLL
β_1AR 371-390	DFRKAFQRLLCCARRAACRR
β_1AR 381-400	CCARRAACRRRAAHGDRPRA
β_1AR 391-410	RAAHGDRPRASGCLARAGPP
β_1AR 401-420	SGCLARAGPPPSPGAPSDDD
β_1AR 411-430	PSPGAPSDDDDDDAGTTPPA
β_1AR 421-440	DDDAGTTPPARLLEPWTGCN
β_1AR 431-450	RLLEPWTGCNGGTTTVDSDS
β_1AR 441-460	GGTTTVDSDSSLDEPGRQGF
β_1AR 451-466	SLDEPGRQGFSSESKV

†peptides could not be synthesized
