# Supplementary material for: β1-Adrenergic Receptor Contains Multiple IAk and IEk Binding Epitopes That Induce T Cell Responses with Varying Degrees of Autoimmune Myocarditis in A/J Mice
Source: Front Immunol. 2017 Nov 20;8:1567. doi: 10.3389/fimmu.2017.01567 (PMC5701947; doi:10.3389/fimmu.2017.01567)
Supplement: Supplementary file 3 [file Table_3.PDF]

Table S3. Identification of pools of β_1AR peptides that are capable of inducing myocarditis with or without T cell responses in A/J mice.

		Myocarditis		T cell responses (fold†)	
Pools	Peptides	Incidence (%)	Number of foci	10 µg/ml	100 µg/ml
	RNase 43-56 (control)			1.05	1.42
	β_1AR 1-20			1.52	1.90
	**β_1AR 11-30**	0	0	**1.84**	**2.06**
I	β_1AR 21-40			1.33	1.43
	β_1AR 31-50			0.97	1.30
	β_1AR 41-60			0.84	1.10
	RNase 43-56 (control)			1.06	1.20
	β_1AR 51-70			1.10	1.38
	**β_1AR 71-90**	0	0	**2.71**	**3.78**
II	β_1AR 81-100			1.65	2.36
	β_1AR 91-110			1.67	1.93
	BAR 101-120			1.01	1.02
	RNase 43-56 (control)			1.04	1.14
	**β_1AR 111-130**			**1.97**	**2.49**
	β_1AR 121-140	0	0	1.08	1.26
III	β_1AR 131-150			1.28	1.72
	**β_1AR 151-170**			**2.24**	**2.43**
	β_1AR 161-180			1.03	1.29
	RNase 43-56 (control)			1.06	1.15
	**β_1AR 171-190**			**2.68**	**5.13**
	**β_1AR 181-200**	1/5 (20)	42	**1.70**	**2.30**
IV	**β_1AR 191-210**			**1.26**	**1.63**
	β_1AR 201-220			1.08	1.37
	β_1AR **211-230**			**3.15**	**3.27**
	RNase 43-56 (control)			1.06	1.14
	β_1AR 221-240			1.18	1.01
	**β_1AR 231-250**	0	0	**1.42**	**1.85**
V	β_1AR 241-260			1.52	2.02
	β_1AR 251-270			1.12	1.81
	β_1AR 261-280			0.86	1.32
	RNase 43-56 (control)			0.73	1.10
	β_1AR 271-290			1.05	1.13
	β_1AR 281-300	0	0	0.92	0.93
VI	β_1AR 291-310			0.87	1.66
	**β_1AR 301-320**			**2.03**	**1.93**
	β_1AR 311-330			1.30	1.21
	RNase 43-56 (control)			1.16	1.16
	**β_1AR 321-340**			**3.23**	**2.21**
	β_1AR 331-350	1/5 (20)	2	1.05	1.49
VII	β_1AR 341-360			1.02	1.47
	**β_1AR 351-370**			**1.86**	**1.82**
	**β_1AR 361-380**			**2.06**	**1.82**
	RNase 43-56 (control)			0.91	0.81
	β_1AR 371-390			1.14	0.60
	β_1AR 381-400	2/5 (40)	20	1.10	1.45
VIII	β_1AR 391-410			1.03	1.18
	β_1AR 401-420			0.90	0.88
	β_1AR 411-430			0.93	0.94
	RNase 43-56 (control)			0.80	0.83
	β_1AR 421-440			1.12	1.21
IX	β_1AR 431-450	2/5 (40)	5	1.14	1.18
	β_1AR 441-460			0.79	1.01
	β_1AR 451-466			0.95	1.16

†Represents fold difference in T cell responses induced by the indicated peptide as compared to medium control. β_1AR peptides that induced significant T cell responses are shown in bold ($P<0.05$).
