# Supplementary material for: β1-Adrenergic Receptor Contains Multiple IAk and IEk Binding Epitopes That Induce T Cell Responses with Varying Degrees of Autoimmune Myocarditis in A/J Mice
Source: Front Immunol. 2017 Nov 20;8:1567. doi: 10.3389/fimmu.2017.01567 (PMC5701947; doi:10.3389/fimmu.2017.01567)
Supplement: Supplementary file 4 [file Table_4.PDF]

Table S4. Myocarditis induced by selected β_1AR peptides.

	Incidence (%)	Inflammatory foci†
β_1AR 171-190	0/5 (0)	0
β_1AR 181-200	1/5 (20)	2
β_1AR 191-210	0/5 (0)	0
β_1AR 201-220	0/5 (0)	0
β_1AR 211-230	2/5 (40)	2.0 ± 1.0
β_1AR 381-400	0/5 (0)	0
β_1AR 391-410	0/5 (0)	0

†Represents mean ± SEM values derived from myocarditic animals
