# Supplementary material for: β1-Adrenergic Receptor Contains Multiple IAk and IEk Binding Epitopes That Induce T Cell Responses with Varying Degrees of Autoimmune Myocarditis in A/J Mice
Source: Front Immunol. 2017 Nov 20;8:1567. doi: 10.3389/fimmu.2017.01567 (PMC5701947; doi:10.3389/fimmu.2017.01567)
Supplement: Supplementary file 5 [file Table_5.PDF]

Table S5. Echocardiographic assessment of cardiac abnormalities in mice immunized with a cocktail of β_1AR_{Ac} 171-190, 181-200 and 211-230.

Parameters	Naïve mice	Immunized mice
Heart rate (beats/min)	337.67 ± 20.98	384.00 ± 10.10
Interventricular septal thickness end-diastole index (mm/BSA)	0.10 ± 0.01	0.12 ± 0.01
Left ventricular internal diameter end-diastole index (mm/BSA)	0.50 ± 0.01	0.45 ± 0.01
Left ventricular posterior wall thickness at end-diastole index (mm/BSA)	0.10 ± 0.02	0.10 ± 0.01
Left ventricular internal diameter end-systole index (mm/BSA)	0.33 ± 0.03	0.26 ± 0.01*
End-diastolic volume (µl)	101.67 ± 3.33	87.50 ± 8.66
End-systolic volume (µl)	33.33 ± 8.33	17.50 ± 2.89
Ejection fraction (%)	67.88 ± 8.08	80.13 ± 2.07
Fractional shortening (%)	33.39 ± 5.61	42.86 ± 2.05
Stroke volume (µl)	66.67 ± 8.33	70.00 ± 8.16
Left ventricular mass (at diastole) index (kg/BSA)	0.95 ± 0.01	0.92 ± 0.004*
Relative wall thickness	0.39 ± 0.06	0.43 ± 0.04

Data represents mean ± SEM values, and *P<0.05 vs naïve group.
