# Supplementary material for: β1-Adrenergic Receptor Contains Multiple IAk and IEk Binding Epitopes That Induce T Cell Responses with Varying Degrees of Autoimmune Myocarditis in A/J Mice
Source: Front Immunol. 2017 Nov 20;8:1567. doi: 10.3389/fimmu.2017.01567 (PMC5701947; doi:10.3389/fimmu.2017.01567)
Supplement: Supplementary file 6 [file Table_6.PDF]

Table S6. Comparison of amino acid sequences of T cell epitopes of mouse β_1AR between different species.

Species	Epitope	Sequence	Identity (%)
		β_1AR 171-190	
Mouse	β_1AR 171-190	TRARARALVCTVWAISALVS	
Human	β_1AR 171-190	TRARAR**<u>G</u>**LVCTVWAISALVS	95
Rat	β_1AR 171-190	TRARARALVCTVWAISALVS	100
Rabbit	β_1AR 218-237	TRARARALVCTVWAISALVS	100
		β_1AR 181-200	
Mouse	β_1AR 181-200	TVWAISALVSFLPILMHWWR	
Human	β_1AR 181-200	TVWAISALVSFLPILMHWWR	100
Rat	β_1AR 181-200	TVWAISALVSFLPILMHWWR	100
Rabbit	β_1AR 228-247	TVWAISALVSFLPILMHWWR	100
		β_1AR 211-230	
Mouse	β_1AR 211-230	NDPKCCDFVTNRAYAIASSV	
Human	β_1AR 211-230	NDPKCCDFVTNRAYAIASSV	100
Rat	β_1AR 211-230	NDPKCCDFVTNRAYAIASSV	100
Rabbit	β_1AR 258-277	NDPKCCDFVTNRAYAIASSV	100

Non-identical residue is bolded and underlined
