# Supplementary material for: β1-Adrenergic Receptor Contains Multiple IAk and IEk Binding Epitopes That Induce T Cell Responses with Varying Degrees of Autoimmune Myocarditis in A/J Mice
Source: Front Immunol. 2017 Nov 20;8:1567. doi: 10.3389/fimmu.2017.01567 (PMC5701947; doi:10.3389/fimmu.2017.01567)
Supplement: Supplementary file 7 [file Table_7.PDF]

Table S7. Evaluation of non-cardiac tissues for inflammatory changes in animals immunized with β_1AR_{Ac} peptides.

Groups	Brain		Lung		Liver		Kidney	
	Incidence (%)	Inflammatory foci	Incidence (%)	Inflammatory foci	Incidence (%)	Inflammatory foci	Incidence (%)	Inflammatory foci
Naïve	0/5 (0)	0	2/5 (40)	0.60 ± 0.40	5/5 (100)	2.20 ± 0.49	0/5 (0)	0
CFA/PT	0/5 (0)	0	4/4 (100)	4.00 ± 0.40	5/5 (100)	19.00 ± 4.60	0/5 (0)	0
β_1AR_{Ac} 171-190	0/6 (0)	0	6/6 (100)	4.33 ± 0.21	6/6 (100)	29.17 ± 6.83	0/6 (0)	0
β_1AR_{Ac} 181-200	0/6 (0)	0	6/6 (100)	3.17 ± 0.60	6/6 (100)	44.50 ± 11.66**	0/6 (0)	0
β_1AR_{Ac} 211-230	0/5 (0)	0	5/6 (83)	2.50 ± 1.34	6/6 (100)	20.33 ± 4.83	0/6 (0)	0
β_1AR_{Ac} 171-190, 181-200 and 211-230	0/5 (0)	0	3/5 (60)	4.20 ± 1.71	5/5 (100)	35.40 ± 8.32*	0/5 (0)	0

Data represents mean ± SEM values with *P<0.05 and **P<0.01 vs naïve group.
