# Supplementary material for: β1-Adrenergic Receptor Contains Multiple IAk and IEk Binding Epitopes That Induce T Cell Responses with Varying Degrees of Autoimmune Myocarditis in A/J Mice
Source: Front Immunol. 2017 Nov 20;8:1567. doi: 10.3389/fimmu.2017.01567 (PMC5701947; doi:10.3389/fimmu.2017.01567)
Supplement: Supplementary file 8 [file Table_8.PDF]

Table S8. Comparison of amino acid sequences between different subtypes of βARs.

Isoform	Sequence	Identity (%)
	**βAR 171-190**	
β_1AR	TRARARALVCTVWAISALVS	
β_2AR	TKNKARVVILMVWIVSGLTS	40
β_3AR	TKRRARAAVVLVWIVSAAVS	60
	**βAR 181-200**	
β_1AR	TVWAISALVSFLPILMHWWR	
β_2AR	MVWIVSGLTSFLPIQMHWYR	65
β_3AR	LVWIVSAAVSFAPIMSQWWR	60
	**βAR 211-230**	
β_1AR	NDPKCCDFVTNRAYAIASSV	
β_2AR	TEETCCDFFTNQAYAIASSI	65
β_3AR	SNPRCCSFASNMPYALLSSS	45

Identical residues are underlined
