# Supplementary figures and images for: β1-Adrenergic Receptor Contains Multiple IAk and IEk Binding Epitopes That Induce T Cell Responses with Varying Degrees of Autoimmune Myocarditis in A/J Mice
Source: Front Immunol. 2017 Nov 20;8:1567. doi: 10.3389/fimmu.2017.01567 (PMC5701947; doi:10.3389/fimmu.2017.01567)

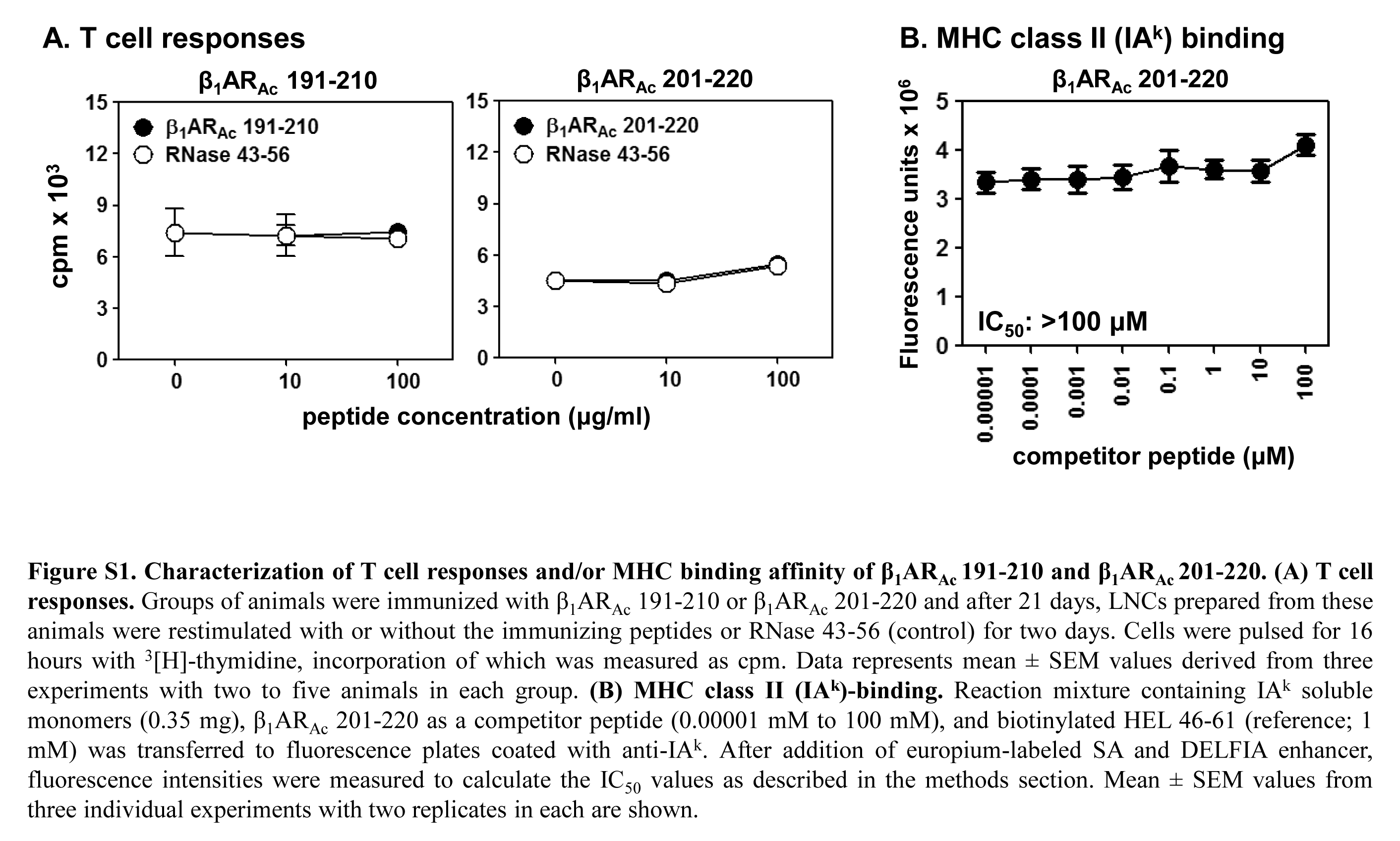

Supplement: Supplementary file 9 [file Image_1.tif]

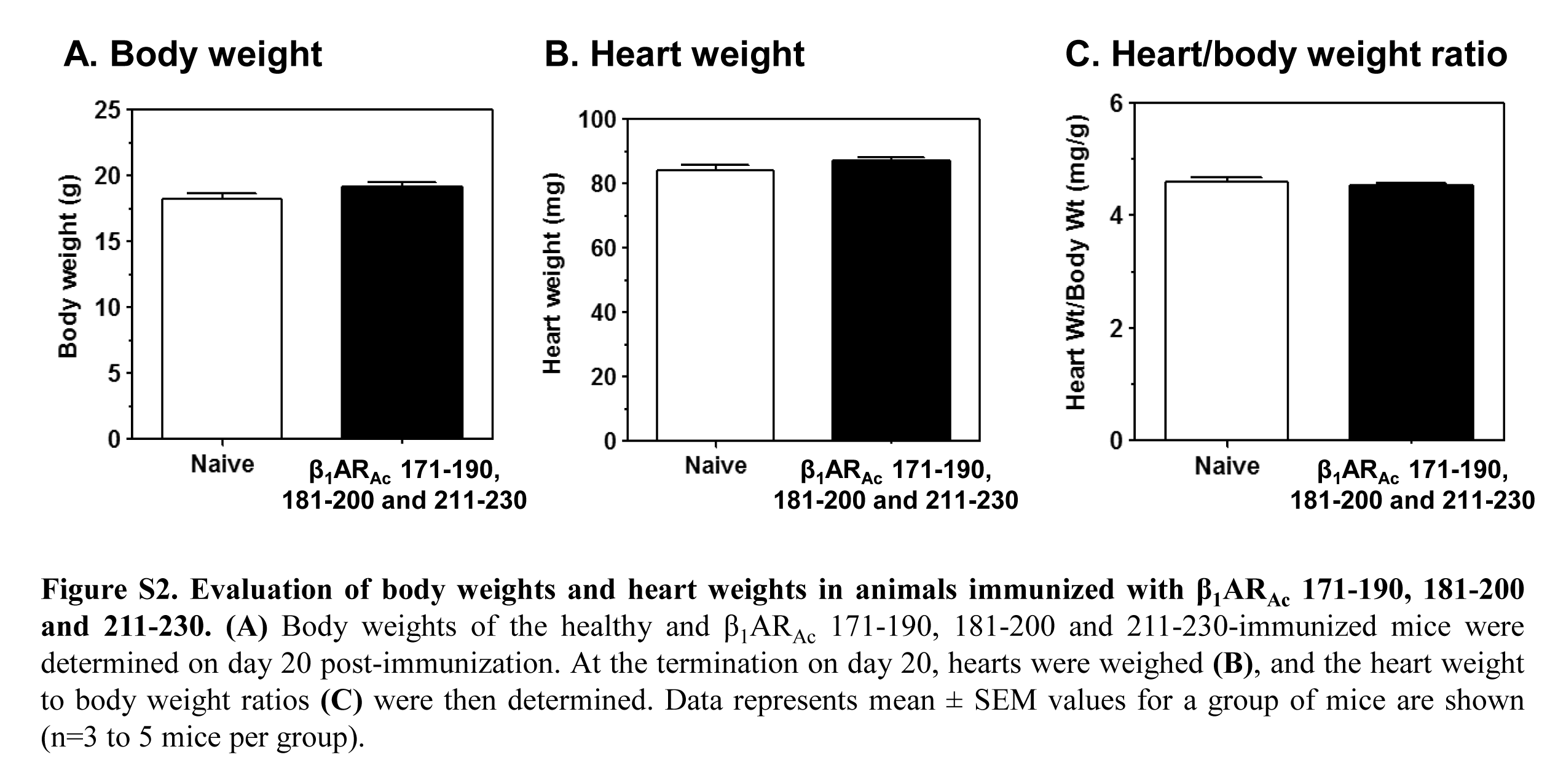

Supplement: Supplementary file 10 [file Image_2.tif]

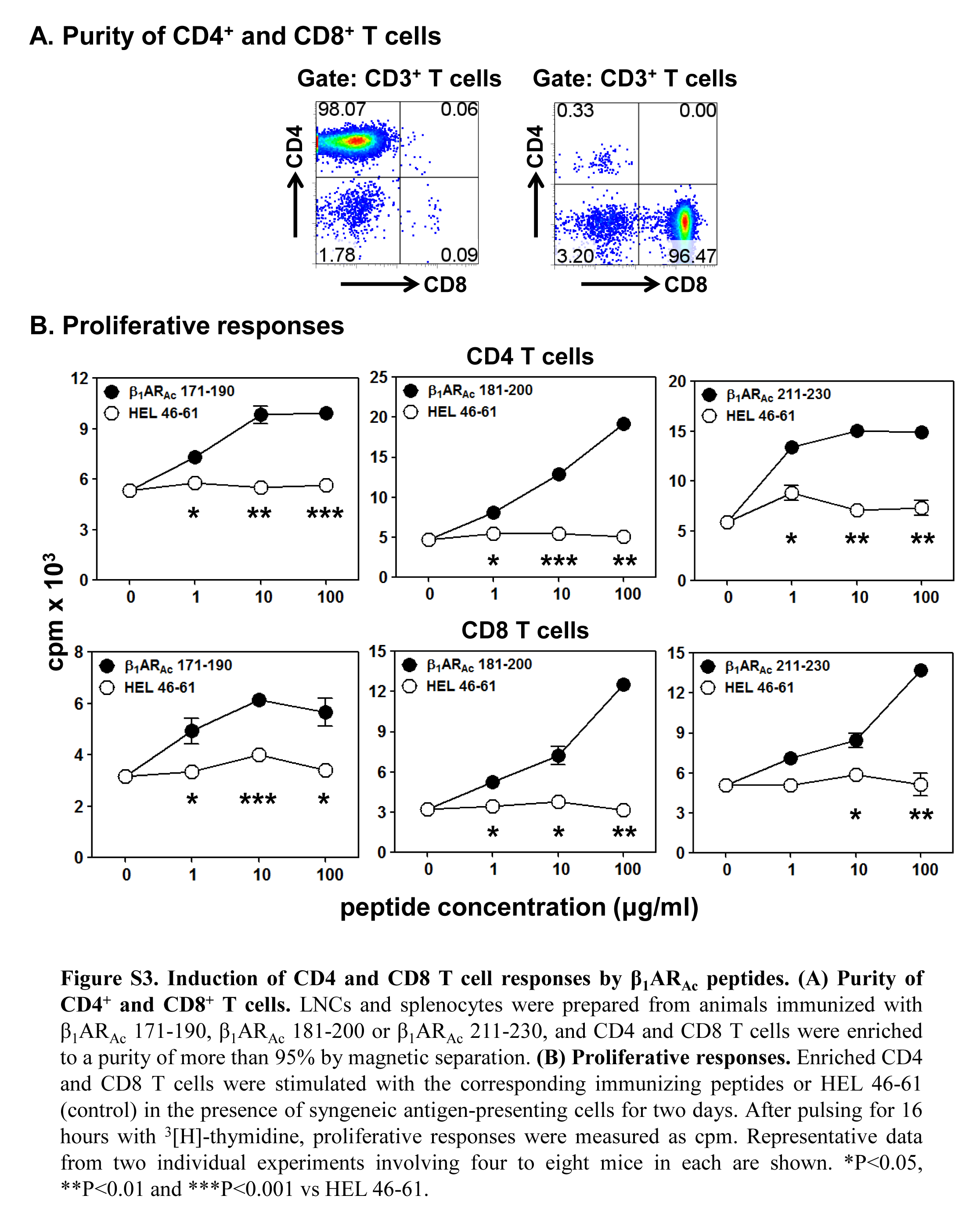

Supplement: Supplementary file 11 [file Image_3.tif]

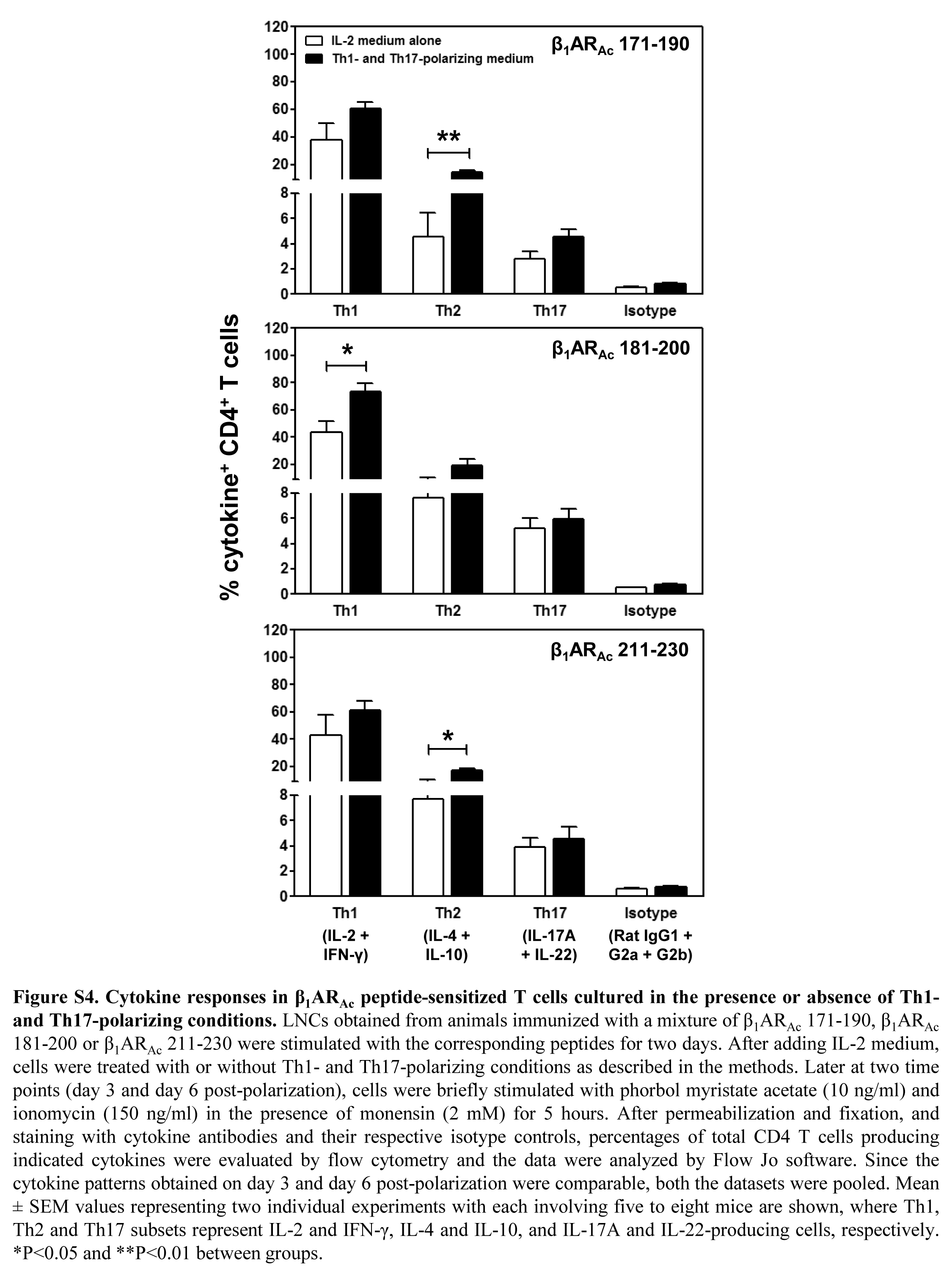

Supplement: Supplementary file 12 [file Image_4.tif]

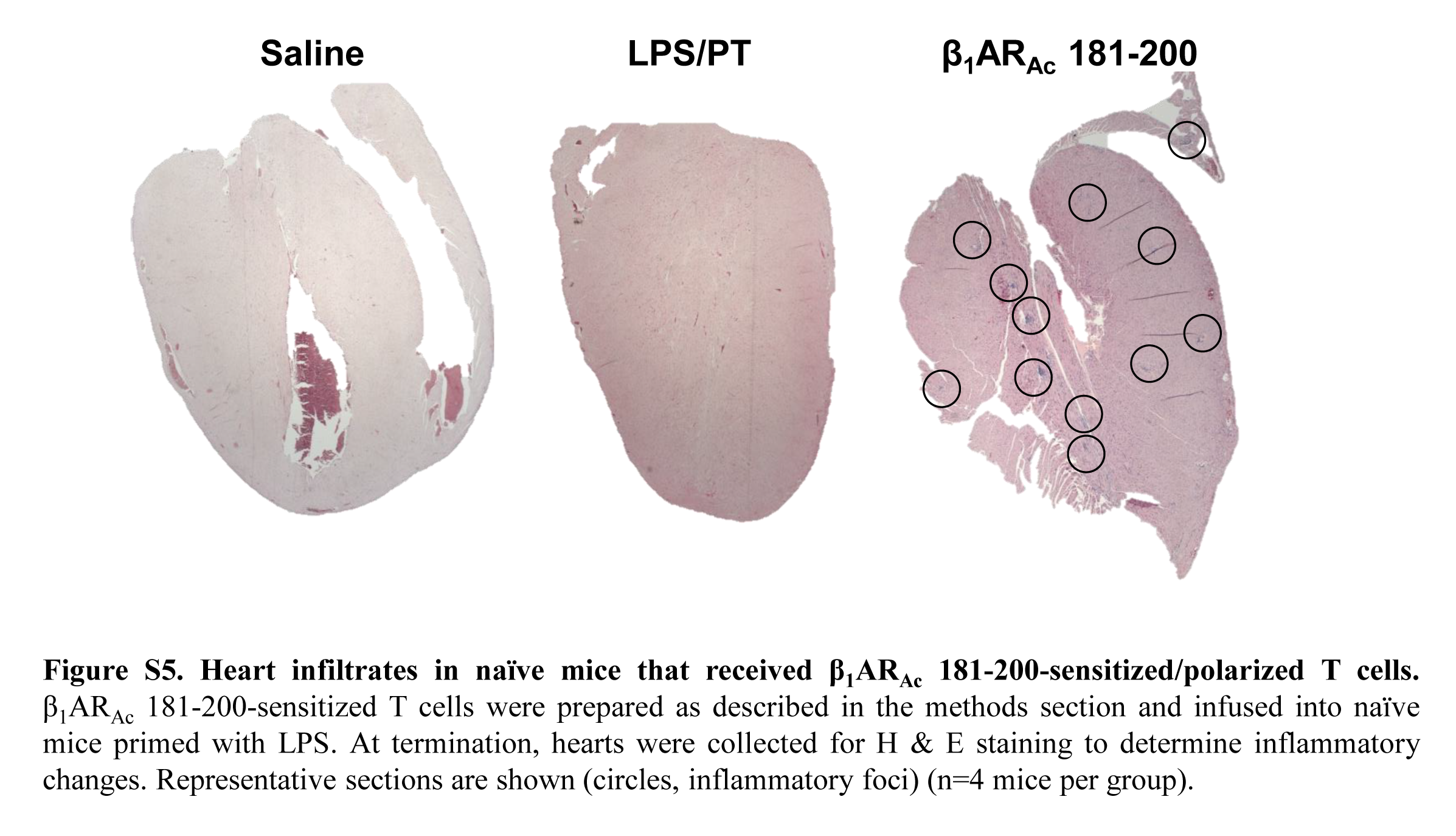

Supplement: Supplementary file 13 [file Image_5.tif]

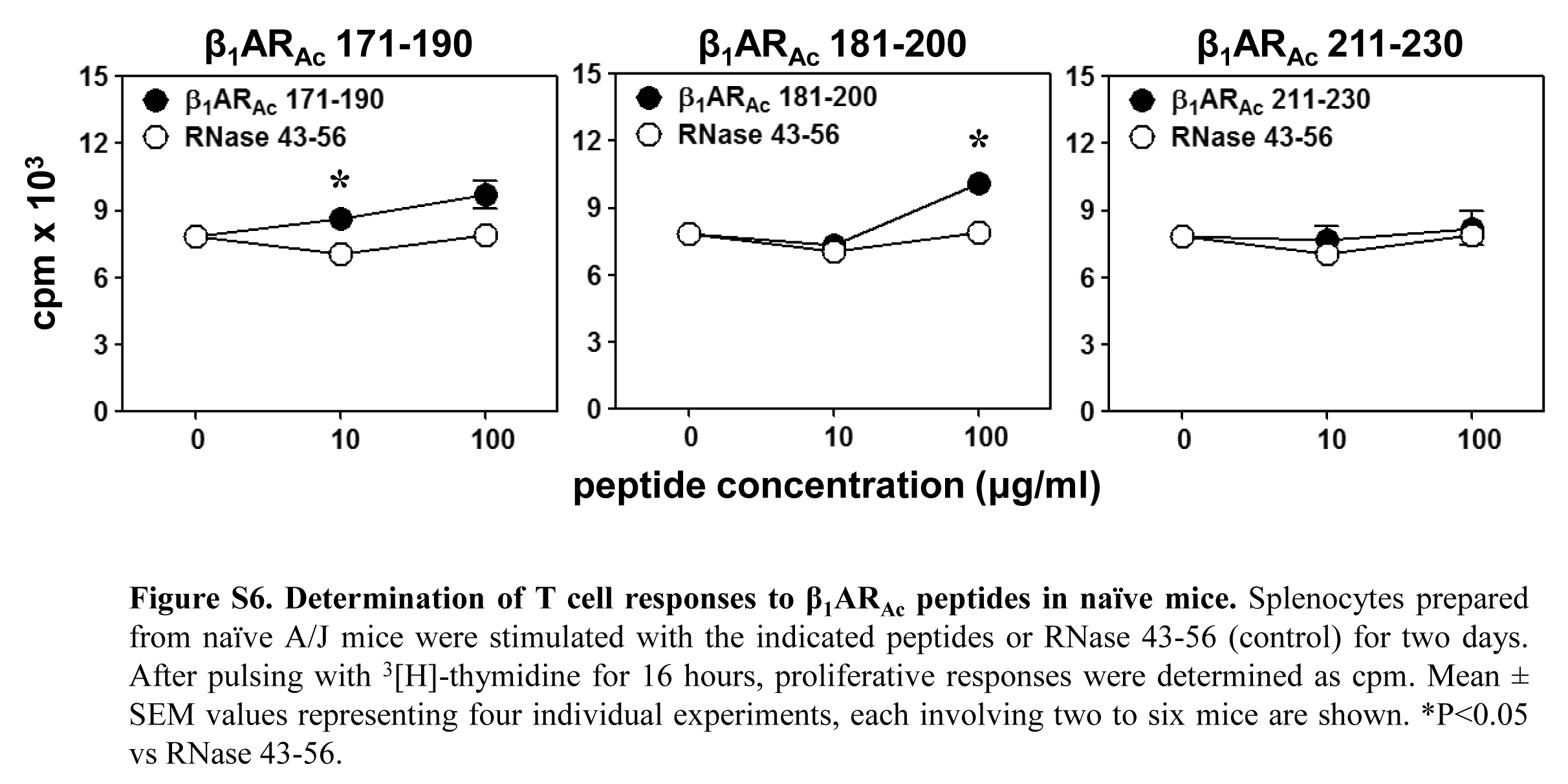

Supplement: Supplementary file 14 [file Image_6.tif]
